# An Optimized Method for Accurate Fetal Sex Prediction and Sex Chromosome Aneuploidy Detection in Non-Invasive Prenatal Testing

**DOI:** 10.1371/journal.pone.0159648

**Published:** 2016-07-21

**Authors:** Ting Wang, Quanze He, Haibo Li, Jie Ding, Ping Wen, Qin Zhang, Jingjing Xiang, Qiong Li, Liming Xuan, Lingyin Kong, Yan Mao, Yijun Zhu, Jingjing Shen, Bo Liang, Hong Li

**Affiliations:** 1 Center for Reproduction and Genetics, Nanjing Medical University Affiliated Suzhou Hospital, Suzhou, Jiangsu, 215002, China; 2 School of Life Sciences & Biotechnology, Shanghai Jiao Tong University, Shanghai, China; 3 Basecare Medical Device Co., Ltd., Suzhou, Jiangsu, China; Warwick University, UNITED KINGDOM

## Abstract

Massively parallel sequencing (MPS) combined with bioinformatic analysis has been widely applied to detect fetal chromosomal aneuploidies such as trisomy 21, 18, 13 and sex chromosome aneuploidies (SCAs) by sequencing cell-free fetal DNA (cffDNA) from maternal plasma, so-called non-invasive prenatal testing (NIPT). However, many technical challenges, such as dependency on correct fetal sex prediction, large variations of chromosome Y measurement and high sensitivity to random reads mapping, may result in higher false negative rate (FNR) and false positive rate (FPR) in fetal sex prediction as well as in SCAs detection. Here, we developed an optimized method to improve the accuracy of the current method by filtering out randomly mapped reads in six specific regions of the Y chromosome. The method reduces the FNR and FPR of fetal sex prediction from nearly 1% to 0.01% and 0.06%, respectively and works robustly under conditions of low fetal DNA concentration (1%) in testing and simulation of 92 samples. The optimized method was further confirmed by large scale testing (1590 samples), suggesting that it is reliable and robust enough for clinical testing.

## Introduction

Since cell-free fetal DNA (cffDNA) was detected in cell-free DNA (cfDNA) of pregnant women plasma by Lo *et al*. in 1997 [1], comparison of chromosome dosage distribution of cffDNA between patient and control plays an increasingly important role in fetal aneuploidy diagnosis. The non-invasive prenatal testing (NIPT) is based on shotgun massively parallel sequencing technology and has been widely applied to detect trisomy 21, 18, 13 and sex chromosome aneuploidies (SCAs). SCAs are characterized by an abnormal number of sex chromosomes and has been linked to a series of diseases, such as Turner syndrome (monosomy X, 1/2000 in baby), Klinefelter syndrome (XXY, 1/500 in male), Triple X syndrome (XXX, 1/2000 in female) and Jacob syndrome (XYY, 1/1000 in male) [2, 3]. The clinical symptoms of SCAs include short stature, a webbed neck, mental disorder and development abnormal development in sexual and intelligence [4]. Unfortunately, recent studies suggested that the NIPT achieved lower accuracy in SCAs detection than autosomal aneuploidies detection [5, 6]. One of the most important reasons is that SCA detection is highly dependent on fetus sex prediction. Chiu, Akolekar *et al* [5] reported that the FNR and FPR of fetal sex prediction were 0.52% and 0.82%, respectively when using an 8-plex sequencing protocol to test pregnant woman with 386 male fetuses and 365 female fetuses. Using a 2-plex sequencing protocol, the FNR and FPR were similar, 0.51% and 0.85% respectively (by testing pregnant woman with 196 male fetuses and 117 female fetuses) [5]. Xiaoyu Pan *et al* analyzed pregnant woman with 423 male fetuses and 377 female fetuses, and the FNR and FPR of fetal sex prediction were 0.47% and 0.8%respectively [6]. The similar results from the two groups indicate that there are additional technology challenges, reducing the accuracy of the fetal sex prediction.

In previous reports [5–8], the proportion of the Y chromosome was considered as a major parameter for fetal sex prediction. This is a ratio of the count of uniquely mapped reads (UMRs) in the Y chromosome divided by the count of UMRs in all chromosomes (autosomal and sex chromosomes). If the percentage of the Y chromosome is higher than the reference (cut-off value), then the sex of the fetus is predicted to be male. However, if a normal female fetus has been predicted to be male by NIPT, she may in fact have Klinefelter syndrome (XXY). Thus means that the fetal sex prediction result directly influences the result of the SCAs detection.

**Fig 1:**
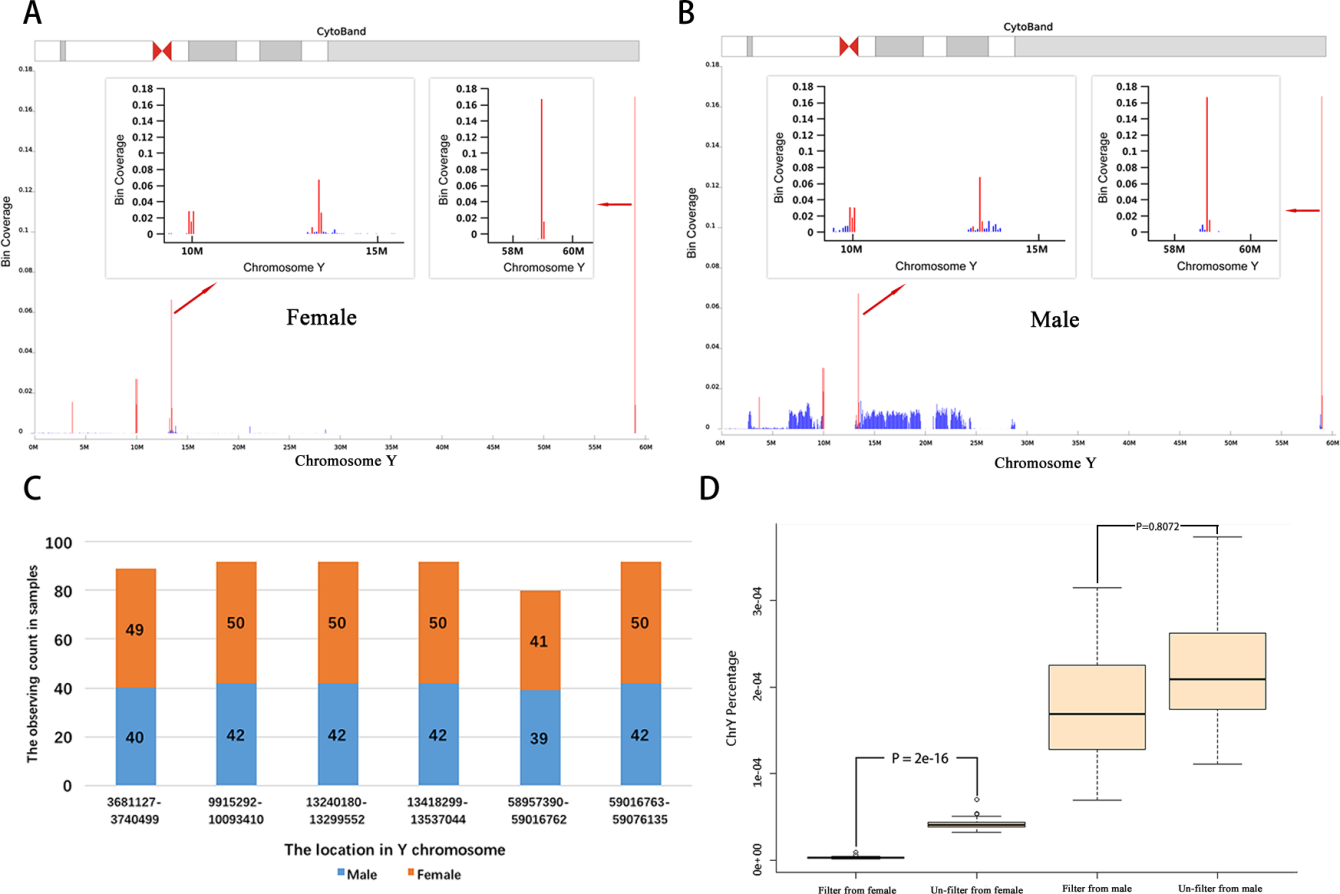
Discovery of six special regions in Y chromosome. A and B show the six highly covered regions (highlighted by red line) in Y chromosome in female and male group. They were located at the same positions of Y chromosome in two groups. C) There are detection counts of six regions in Y chromosome of 92 samples. D) The comparison of Y chromosome reads percentage in the female and male fetus groups in before and after filtering out the reads in six regions of Y chromosome.

To explore the reasons for high FNR and FPR of fetal sex prediction, we collected 92 plasma samples from pregnant women having 50 female fetuses and 42 male fetuses, followed the standard protocol of sequencing. After comparing the cover ranges of UMRs in the Y chromosome between the male and female groups, we found that some UMRs are highly enriched in six regions of the Y chromosome. Interestingly, these UMRs may be observed in both the female and male groups (Fig 1A, 1B and 1C). This result indicates that those UMRs may not be mapped correctly in both these groups and therefore result in higher FNR and FPR in fetal sex prediction. To deal with this problem, these problematic UMRs were filtered out and the DNA concentrations were then recalculated as regards fetal sex prediction. This lead to a significant decrease in the FNR and FPR (from nearly 1% to 0.01% and 0.06%) of fetal sex prediction, even when the fetal DNA concentration is 1%. The improved effectiveness of our optimized method was further confirmed in large-scale testing of clinical samples (1590 samples) using the method reported by Chiu *et*.*al* [5] as a control to evaluate the improvement of our method.

## Materials and Methods

### Sample collection and sequencing

In this study, 92 pregnant women were recruited with plasma collected after informed consent. These women were more than 20 years old with singleton pregnancy. The gestational ages were at least 15 weeks (Table 1). The fetal sex was known via follow-up call after delivery (50 females and 42 males). All plasma samples were processed and sequenced according to the standard protocol of Ion Proton [7]. Briefly, cell-free DNA was extracted from 600μl plasma with the TIANamp Micro DNA Purification Kit (Tiangen Biotech) and the sequencing library prepared following the manufacturer's instructions. Then the sequencing library was loaded onto an Ion P2 chip. A standard 30-cycle of Ion torrent sequencing was run in a single-end sequencing model. The primary sequencing data were processed by the IonTorrent platform-specific pipeline software (Torrent Suite, version 2.0.1) in order to generate sequence reads, to trim adapter sequences and filter out low-quality reads. The initial data processing of the 92 samples corresponded to that of the 1590 clinical samples (794 female and 796 male fetuses).

**Table 1:** Basic information of 92 samples of pregnant women.

Maternal Age (years)		Gestational Age (weeks)		Sequencing reads number	
Range	Mean±SD	Range	Mean±SD	Mean (original)	Mean (unique)
20–41	29.9±5.6	15–21	16.8±1.3	7,644,385	4,950,761
**Sample Number**		92			

SD: standard deviation.

### Data analysis

All sequencing data were mapped to the human reference genome of hg19 (version: NCBI Build37/hg19) by bowtie2 software [9] and four types of mapped reads: PCR duplicates, short reads (short than 35 bp), multi-mapped reads and low quality reads (MAPQ score < 10) were removed by a perl script [10]. The percentage of reads mapped to each chromosome was calculated using the number of UMRs in a selected chromosome, divided by the count of UMRs in all chromosomes (autosomal and sex chromosomes) after normalizing the number of the uniquely mapping reads by LOESS regression for execution GC correction [10]. The corrected and original Y chromosome percentages were calculated using corrected UMRs number (filtering out UMRs at the six special regions in the Y chromosome) and original UMRs number (not filtering out UMRs at the six special regions in the Y chromosome), respectively.

### Calculation of the male fetal DNA concentration

Measuring fetal DNA concentration accurately is essential for NIPT, because it was used to calculate the percentage of the Y for male fetuses, as a baseline cutoff to determine the fetal sex. The formula we used to calculate fetal DNA concentration was reported by Chiu *et*.*al* [5].

$$\mathrm{Fetal DNA Concentration}\;(F)=\frac{\%chrY_{MF}-\%chrY_{FF}}{\%chrY_{AM}-\%chrY_{FF}}$$

Here, %chrY_MF_ is the Y chromosome percentage of pregnant women carrying a male fetus; %chrY_FF_ and %chrY_AM_ are the median of the Y chromosome percentage from pregnant women and three adult males. The values of the %chrY_FF_ and the %chrY_AM_ were calculated twice, before and after filtering out reads mapped to six special regions of the Y chromosome. The results were then used to calculate the two values of %chrY_MF_ before and after filtering out reads mapped to the six special regions, when assuming that the fetal DNA concentration was equal to 1%. The value of %chrY_FF_ was the median of the Y chromosome percentage from 50 pregnant women with female fetuses in 92 samples. These values are listed in Table 2.

**Table 2:** The value for calculation of fetal DNA concentration.

%chrY_FF (median)		%chrY_AM (median)		%chrY_MF				Fetal DNA concentration region	
Original method	Optimized method	Original method	Optimized method	Minimum in original method	Maximum in optimized method	Minimum in original method	Maximum in optimized method	Original method	Optimized method
0. 0000422	0.00000302	0.00173	0.000374	0.000111	0.00170	0.0000727	0.000315	4.1% ~ 19.69%	4.1% ~ 18.38%
The %chrY_MF value when assumption fetal DNA concentration is 1%									
Original method		F = 1%	%chrY_FF (median) = 0. 0000422		%chrY_AM (median) = 0.00173		%chrY_MF = 0.0000591		
Optimized method		F = 1%	%chrY_FF (median) = 0.00000302		%chrY_AM (median) = 0.000374		%chrY_MF = 0.00002		

## Results

### Identification of six randomly mapped regions in the Y chromosome

The sequencing data from the 92 samples were processed and classified into two groups, based on known fetal sex: female and male. The UMRs counts in the 92 samples are in the range of 2,511,824 to 7,392,092, after executing GC correction. Using the ordinary methods [8, 11], the ratio of the minimal percentage of chromosome Y in the male group to the maximal percentage of chromosome Y in female group is only 1.58 (S1 Table). The small variation of the Y chromosome percentages between females and males may be a potential reason for higher FNR and FPR in fetal sex detection.

We compared the mapping patterns of the reads (the distribution of UMRs) of the Y chromosome between the male and female groups, discovering six regions that always have extraordinary higher coverage depth than the average in both groups (Fig 1A, 1B and 1C). More importantly, about 92% of UMRs in the Y chromosome were mapped in these same regions in the female group, compared to 20% in the male group (Tables 3 and 4). These results suggest that these reads may not come from the Y chromosome but were randomly mapped in the female, because 1) DNA fragments from the Y chromosome should not be observed in sequencing data from the female; 2) the abundant variations and repeat DNA sequences in the Y chromosome could result in random mapping for short sequencing reads. To increase the accuracy of fetal sex prediction, the UMRs at six regions of the Y chromosome were filtered out in both the male and female groups. As a result, the ratio of the minimal percentage of the Y chromosome in the male group to the maximal percentage of chromosome Y in the female group increased from 1.58 to 7.96. Statistical analysis also indicated that the difference of the Y chromosome percentage is significant between the female and male groups (p value: 0.8 vs 2E-16) (Fig 1D).

**Table 3:** The calculated percentage of Y chromosome using original and optimized methods.

Fetal gender	Original method		Optimized method		Mean Rate (Optimized/Original)
	Range	Mean±SDa	Range	Mean±SDa	
**Male**	1.11E-04 ~ 3.74E-04	2.22E-04 ±6.98E-05	6.95E-05 ~ 3.15E-04	1.78E-04 ±6.72E-05	80%
**Female**	3.24E-05 ~ 7.02E-05	4.22E-05 ±6.15E-06	1.26E-06~9.13E-06	3.02E-06 ±1.37E-06	8%

**Table 4:** The six regions discovered in Y chromosome.

Begin location	End location	Bin Coverage	CytoBand
3,681,127	3,740,499	0.016	p11.2
9,915,292	10,093,410	0.023	p11.2
13,240,180	13,299,552	0.008	q11.1
13,418,299	13,537,044	0.039	q11.21
58,957,390	59,016,762	0.167	q12
59,016,763	59,076,135	0.014	q12

### Method validation under condition of low fetal DNA concentration

**Fig 2:**
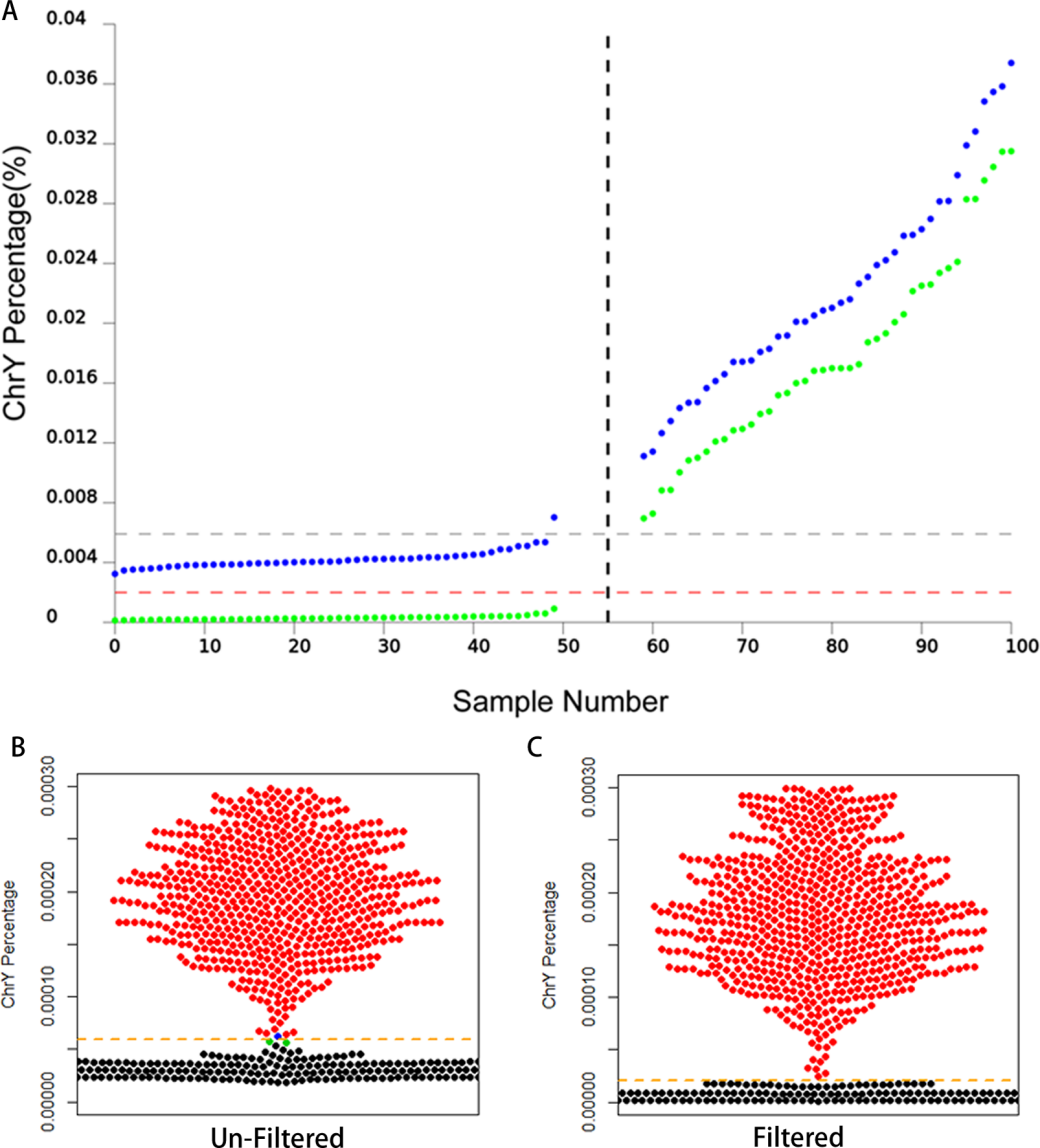
Testing of the optimized method in large-scale sample. A) The DNA percentages of Y chromosome calculated by optimized method and original method. Here, the fetal DNA concentration is assumed as 1%. The percentage of Y chromosome calculated by the optimized method was marked as green point. The blue point illustrates the percentage of Y chromosome calculated by original method. B and C) the percentage of Y chromosome calculated by optimized method and original method in 1590 samples. Here, black and red dots represent the percentage of Y chromosome in female fetuses and male fetus, respectively. Error prediction results were marked by blue and green dots in Fig C in which two male fetuses were predicted to be female and marked by blue; a female fetus predicted to be male and marked by green.

We also compared the performance between the optimized method and the original method, when fetal DNA concentration is low. According to the fetal concentration formula reported by Chiu *et*.*al* [5], we calculated the fetal DNA concentration for the male group, using the value of the Y chromosome percentage, obtained from the optimized method and the original method, respectively. We found that the two methods ended up with similar results as regards the fetal DNA concentration prediction in the male group (optimized method: 4.1% ~ 19.69%; original method: 4.1% ~ 18.38%). According to previous research the fetal DNA concentration is in the range of 1.6% ~ 40% [12], which means that the cut-off value (%chrY_MF_) for detection of fetal sex is appropriate if the male fetal DNA concentration is lower than 1.6%. The NIPT guideline of ACOG (2015) also suggested that the cffDNA fraction is generally between 3% and 13% in total cell-free maternal DNA after 10 weeks of gestation and 10% or more may have a fetal fraction of less than 4%. It indicates that the heterogeneity between pregnant women result in the significant difference in cffDNA concentration, such as excessive fat (over 250 pounds)[13]. Based on these, we have chosen 1% as the male fetal DNA concentration to calculate the %chrY_MF_ by the optimized method and the original method respectively, giving the %chrY_MF_ value of the optimized method and the original method as 0.00002 and 0.0000591 respectively (Table 2). Based on the cut-off values (0.00002 and 0.0000591), the fetal sex of 92 samples were re-predicted and a false negative result was observed in the original results (blue dots) but none was found in the result of the optimized method (green dots) (Fig 2A). These results indicate that the FNR of the original method was over 1% in fetal sex detection. To estimate the robustness of the optimized method, we collected 1590 clinical samples to compare the FNR and FPR of the optimized method and the original method. The result demonstrated that one false positive case (FPR nearly 1/1600) and 2 false negative cases (FNR nearly 1/795) were reported in the results processed by the original method but none in the optimized results (Fig 2B and 2C and S2 Table). This indicated that the optimized method was more accurate in fetal sex detection than the original method. In 1590 samples seven Turner syndrome (XO) fetuses were detected by both the optimized and the original method. They were considered to be female, because the percentage of X chromosomes in the Turner syndrome is significantly lower than in the normal female. The result indicated that the optimized method had an equivalent sensitivity in comparison to the original and was significantly better than the original one in fetal sex prediction, in particular when DNA fetal DNA concentration is low.

## Discussion

Non-invasive prenatal testing has been widely used to detect trisomy 21, 18, 13 and sex chromosome aneuploidies (SCAs). The result of fetal sex prediction is dependent on SCAs detection. However, the current data processing method has some limitations to achieve higher accuracy. In this study, we simply filtering out randomly mapping reads at the six special regions in the Y chromosome to achieve significantly smaller FNR and FPR (from nearly 1% to the low 0.1%) of fetal sex detection than the current method [5, 6]. The robustness of the optimized method was validated in testing of large samples (1590 cases) and under the condition of low fetal DNA concentration (1%). But, the improving calculation method cannot filter out these UMRs, deriving from nested sequences of the abnormal sex chromosome or constitutional variation of the maternal X-chromosome copy number. The chimera couldn’t be detected because some cells in the body have XX and others have XY (46, XX/XY), though it is rare [14, 15]. In this situation, using placenta cells to predict fetal gender and detect sex chromosome aneuploidy were unreliable. Additionally, we also noted that the FNR and FPR of our testing were significantly lower than that reported by Xiaoyu Pan *et al* and Chiu *et al*, even when using the same calculation method (the original method). Further analysis indicated that the variation of FNR and FPR between these studies may derive from the difference of the sequencing platform. Xiaoyu Pan *et al* and Chiu *et al* used the Illumina except Ion Proton platform. One of the major differences between these is the length of the reads. The median reads length of the Ion Proton platform is about 140 bp, which is three times longer than that of the Illumina platform. This may suggest that the longer read length is essential to achieve better FNR and FPR as regards NIPT because longer reads has less a chance to be mapped to the wrong location, when there are regions with high sequence similarity between sex chromosomes and autosomes. Overall, our study presents a method in which an additional data processing step can improve the accuracy of fetal sex detection and SCAs detection significantly.

## Supporting Information

S1 Table The list of uniquely mapped reads percentage of Y chromosome in 92 samples and the fetal DNA concentration in male group. (DOCX)

S2 Table The list of uniquely mapped reads percentage of Y chromosome and sex prediction result in 1590 samples. (DOCX)
