# Supplementary material for: An Optimized Method for Accurate Fetal Sex Prediction and Sex Chromosome Aneuploidy Detection in Non-Invasive Prenatal Testing
Source: PLoS One. 2016 Jul 21;11(7):e0159648. doi: 10.1371/journal.pone.0159648 (PMC4956272; doi:10.1371/journal.pone.0159648)
Supplement: S1 Table — (DOCX) [file pone.0159648.s001.docx]

**S1 Table**: The list of uniquely mapped reads percentage of Y chromosome in 92 samples and the fetal DNA concentration in male group

| Female | | Male | | | |
| --- | --- | --- | --- | --- | --- |
| Percentage of chrY | | Percentage of chrY | | Fetal DNA concentration | |
| Original method | Optimized method | Original method | Optimized method | Original method | Optimized method |
| 3.24E-05 | 1.78E-06 | 1.11E-04 | 7.27E-05 | 4.09% | 4.11% |
| 3.47E-05 | 2.63E-06 | 1.14E-04 | 6.95E-05 | 4.26% | 3.92% |
| 3.53E-05 | 3.33E-06 | 1.27E-04 | 8.85E-05 | 5.00% | 5.04% |
| 3.56E-05 | 1.60E-06 | 1.35E-04 | 8.82E-05 | 5.47% | 5.02% |
| 3.60E-05 | 2.53E-06 | 1.43E-04 | 1.00E-04 | 5.99% | 5.73% |
| 3.64E-05 | 2.05E-06 | 1.47E-04 | 1.08E-04 | 6.20% | 6.20% |
| 3.72E-05 | 1.66E-06 | 1.47E-04 | 1.10E-04 | 6.22% | 6.31% |
| 3.75E-05 | 3.95E-06 | 1.57E-04 | 1.14E-04 | 6.78% | 6.55% |
| 3.82E-05 | 1.84E-06 | 1.61E-04 | 1.22E-04 | 7.06% | 7.04% |
| 3.83E-05 | 1.97E-06 | 1.66E-04 | 1.21E-04 | 7.33% | 6.95% |
| 3.84E-05 | 3.57E-06 | 1.74E-04 | 1.28E-04 | 7.82% | 7.39% |
| 3.86E-05 | 4.04E-06 | 1.74E-04 | 1.32E-04 | 7.83% | 7.62% |
| 3.87E-05 | 2.70E-06 | 1.75E-04 | 1.29E-04 | 7.87% | 7.44% |
| 3.87E-05 | 2.66E-06 | 1.81E-04 | 1.39E-04 | 8.21% | 8.03% |
| 3.88E-05 | 3.15E-06 | 1.83E-04 | 1.41E-04 | 8.34% | 8.13% |
| 3.93E-05 | 2.27E-06 | 1.91E-04 | 1.52E-04 | 8.82% | 8.77% |
| 3.95E-05 | 3.36E-06 | 1.92E-04 | 1.53E-04 | 8.86% | 8.86% |
| 3.96E-05 | 4.07E-06 | 2.01E-04 | 1.70E-04 | 9.41% | 9.83% |
| 3.97E-05 | 2.09E-06 | 2.01E-04 | 1.60E-04 | 9.42% | 9.25% |
| 4.00E-05 | 3.76E-06 | 2.05E-04 | 1.61E-04 | 9.66% | 9.33% |
| 4.02E-05 | 2.41E-06 | 2.09E-04 | 1.70E-04 | 9.86% | 9.84% |
| 4.04E-05 | 1.99E-06 | 2.10E-04 | 1.70E-04 | 9.96% | 9.83% |
| 4.04E-05 | 2.96E-06 | 2.14E-04 | 1.68E-04 | 10.16% | 9.73% |
| 4.06E-05 | 1.26E-06 | 2.16E-04 | 1.69E-04 | 10.30% | 9.76% |
| 4.06E-05 | 1.41E-06 | 2.26E-04 | 1.90E-04 | 10.92% | 10.99% |
| 4.08E-05 | 2.99E-06 | 2.31E-04 | 1.87E-04 | 11.18% | 10.86% |
| 4.15E-05 | 3.13E-06 | 2.39E-04 | 2.06E-04 | 11.65% | 11.96% |
| 4.18E-05 | 1.63E-06 | 2.42E-04 | 2.01E-04 | 11.85% | 11.65% |
| 4.23E-05 | 2.15E-06 | 2.47E-04 | 1.73E-04 | 12.16% | 9.99% |
| 4.23E-05 | 3.17E-06 | 2.58E-04 | 1.93E-04 | 12.81% | 11.21% |
| 4.24E-05 | 4.21E-06 | 2.59E-04 | 2.26E-04 | 12.85% | 13.13% |
| 4.25E-05 | 2.93E-06 | 2.63E-04 | 2.21E-04 | 13.07% | 12.87% |
| 4.25E-05 | 3.99E-06 | 2.70E-04 | 2.25E-04 | 13.48% | 13.09% |
| 4.26E-05 | 1.81E-06 | 2.81E-04 | 2.34E-04 | 14.18% | 13.59% |
| 4.32E-05 | 2.67E-06 | 2.82E-04 | 2.41E-04 | 14.19% | 14.03% |
| 4.35E-05 | 2.52E-06 | 2.99E-04 | 2.37E-04 | 15.21% | 13.78% |
| 4.35E-05 | 1.84E-06 | 3.19E-04 | 2.83E-04 | 16.39% | 16.49% |
| 4.37E-05 | 2.00E-06 | 3.28E-04 | 2.83E-04 | 16.95% | 16.50% |
| 4.43E-05 | 3.50E-06 | 3.48E-04 | 2.96E-04 | 18.14% | 17.24% |
| 4.47E-05 | 3.24E-06 | 3.55E-04 | 3.05E-04 | 18.51% | 17.77% |
| 4.52E-05 | 3.49E-06 | 3.58E-04 | 3.15E-04 | 18.73% | 18.39% |
| 4.56E-05 | 1.84E-06 | 3.74E-04 | 3.15E-04 | 19.66% | 18.37% |
| 4.69E-05 | 3.41E-06 |  |  |  |  |
| 4.88E-05 | 2.70E-06 |  |  |  |  |
| 4.89E-05 | 5.92E-06 |  |  |  |  |
| 5.09E-05 | 4.13E-06 |  |  |  |  |
| 5.11E-05 | 5.85E-06 |  |  |  |  |
| 5.35E-05 | 2.93E-06 |  |  |  |  |
| 5.36E-05 | 4.82E-06 |  |  |  |  |
| 7.02E-05 | 9.13E-06 |  |  |  |  |
