# Supplementary material for: An Optimized Method for Accurate Fetal Sex Prediction and Sex Chromosome Aneuploidy Detection in Non-Invasive Prenatal Testing
Source: PLoS One. 2016 Jul 21;11(7):e0159648. doi: 10.1371/journal.pone.0159648 (PMC4956272; doi:10.1371/journal.pone.0159648)
Supplement: S2 Table — (DOCX) [file pone.0159648.s002.docx]

**S2 Table**: The list of uniquely mapped reads percentage of Y chromosome and sex prediction result in 1590 samples

| Sample ID | Original method | | | Optimized method | | |
| --- | --- | --- | --- | --- | --- | --- |
|  | Percentage of chrY | Sex prediction | Fetal DNA concentration | Percentage of chrY | Sex prediction | Fetal DNA concentration |
| 1590 | 0.000774635 | Male | 43.40% | 7.36E-04 | Male | 43.22% |
| 1589 | 0.000585349 | Male | 32.18% | 5.50E-04 | Male | 32.25% |
| 1588 | 0.000546951 | Male | 29.91% | 5.12E-04 | Male | 30.01% |
| 1587 | 0.000506873 | Male | 27.53% | 4.75E-04 | Male | 27.84% |
| 1586 | 0.000497807 | Male | 26.99% | 4.66E-04 | Male | 27.27% |
| 1585 | 0.000487317 | Male | 26.37% | 4.55E-04 | Male | 26.66% |
| 1584 | 0.000449927 | Male | 24.16% | 4.22E-04 | Male | 24.67% |
| 1583 | 0.000444737 | Male | 23.85% | 4.18E-04 | Male | 24.43% |
| 1582 | 0.000441622 | Male | 23.67% | 4.15E-04 | Male | 24.26% |
| 1581 | 0.000430578 | Male | 23.01% | 4.04E-04 | Male | 23.60% |
| 1580 | 0.000433981 | Male | 23.21% | 4.02E-04 | Male | 23.53% |
| 1579 | 0.00042484 | Male | 22.67% | 3.95E-04 | Male | 23.07% |
| 1578 | 0.000426252 | Male | 22.75% | 3.93E-04 | Male | 22.98% |
| 1577 | 0.00042365 | Male | 22.60% | 3.92E-04 | Male | 22.92% |
| 1576 | 0.000414075 | Male | 22.03% | 3.87E-04 | Male | 22.61% |
| 1575 | 0.000414178 | Male | 22.04% | 3.84E-04 | Male | 22.43% |
| 1574 | 0.000412935 | Male | 21.97% | 3.81E-04 | Male | 22.28% |
| 1573 | 0.000396272 | Male | 20.98% | 3.69E-04 | Male | 21.55% |
| 1572 | 0.000399556 | Male | 21.17% | 3.69E-04 | Male | 21.54% |
| 1571 | 0.000397597 | Male | 21.06% | 3.67E-04 | Male | 21.45% |
| 1570 | 0.000396393 | Male | 20.99% | 3.67E-04 | Male | 21.43% |
| 1569 | 0.000393108 | Male | 20.79% | 3.66E-04 | Male | 21.39% |
| 1568 | 0.000391441 | Male | 20.69% | 3.64E-04 | Male | 21.26% |
| 1567 | 0.000393067 | Male | 20.79% | 3.59E-04 | Male | 20.96% |
| 1566 | 0.000388665 | Male | 20.53% | 3.57E-04 | Male | 20.84% |
| 1565 | 0.000388357 | Male | 20.51% | 3.54E-04 | Male | 20.66% |
| 1564 | 0.000381119 | Male | 20.08% | 3.52E-04 | Male | 20.54% |
| 1563 | 0.000379899 | Male | 20.01% | 3.51E-04 | Male | 20.51% |
| 1562 | 0.000379318 | Male | 19.97% | 3.51E-04 | Male | 20.49% |
| 1561 | 0.000382177 | Male | 20.14% | 3.50E-04 | Male | 20.42% |
| 1560 | 0.000380106 | Male | 20.02% | 3.49E-04 | Male | 20.39% |
| 1559 | 0.000377834 | Male | 19.89% | 3.48E-04 | Male | 20.30% |
| 1558 | 0.000377885 | Male | 19.89% | 3.47E-04 | Male | 20.30% |
| 1557 | 0.000374794 | Male | 19.71% | 3.47E-04 | Male | 20.28% |
| 1556 | 0.000374787 | Male | 19.71% | 3.47E-04 | Male | 20.25% |
| 1555 | 0.000372344 | Male | 19.56% | 3.43E-04 | Male | 20.02% |
| 1554 | 0.000370748 | Male | 19.47% | 3.40E-04 | Male | 19.89% |
| 1553 | 0.00036634 | Male | 19.20% | 3.40E-04 | Male | 19.87% |
| 1552 | 0.000367633 | Male | 19.28% | 3.38E-04 | Male | 19.76% |
| 1551 | 0.000365505 | Male | 19.16% | 3.37E-04 | Male | 19.67% |
| 1550 | 0.000363311 | Male | 19.03% | 3.36E-04 | Male | 19.65% |
| 1549 | 0.000365882 | Male | 19.18% | 3.35E-04 | Male | 19.56% |
| 1548 | 0.000360554 | Male | 18.86% | 3.33E-04 | Male | 19.43% |
| 1547 | 0.000363384 | Male | 19.03% | 3.32E-04 | Male | 19.41% |
| 1546 | 0.000362765 | Male | 18.99% | 3.31E-04 | Male | 19.33% |
| 1545 | 0.000358509 | Male | 18.74% | 3.29E-04 | Male | 19.24% |
| 1544 | 0.000358167 | Male | 18.72% | 3.26E-04 | Male | 19.05% |
| 1543 | 0.000358313 | Male | 18.73% | 3.26E-04 | Male | 19.01% |
| 1542 | 0.000354017 | Male | 18.47% | 3.25E-04 | Male | 19.00% |
| 1541 | 0.000355005 | Male | 18.53% | 3.25E-04 | Male | 18.99% |
| 1540 | 0.000357178 | Male | 18.66% | 3.25E-04 | Male | 18.96% |
| 1539 | 0.000354487 | Male | 18.50% | 3.24E-04 | Male | 18.91% |
| 1538 | 0.000352646 | Male | 18.39% | 3.23E-04 | Male | 18.84% |
| 1537 | 0.000353406 | Male | 18.44% | 3.21E-04 | Male | 18.75% |
| 1536 | 0.000345498 | Male | 17.97% | 3.18E-04 | Male | 18.56% |
| 1535 | 0.000346698 | Male | 18.04% | 3.17E-04 | Male | 18.51% |
| 1534 | 0.000349651 | Male | 18.22% | 3.16E-04 | Male | 18.44% |
| 1533 | 0.000341543 | Male | 17.74% | 3.13E-04 | Male | 18.28% |
| 1532 | 0.000344703 | Male | 17.92% | 3.13E-04 | Male | 18.27% |
| 1531 | 0.000342775 | Male | 17.81% | 3.12E-04 | Male | 18.22% |
| 1530 | 0.000342733 | Male | 17.81% | 3.11E-04 | Male | 18.13% |
| 1529 | 0.000338307 | Male | 17.54% | 3.10E-04 | Male | 18.11% |
| 1528 | 0.000337596 | Male | 17.50% | 3.09E-04 | Male | 18.01% |
| 1527 | 0.00033867 | Male | 17.57% | 3.08E-04 | Male | 17.96% |
| 1526 | 0.000340643 | Male | 17.68% | 3.07E-04 | Male | 17.94% |
| 1525 | 0.000338284 | Male | 17.54% | 3.07E-04 | Male | 17.93% |
| 1524 | 0.000336675 | Male | 17.45% | 3.07E-04 | Male | 17.93% |
| 1523 | 0.000338132 | Male | 17.53% | 3.07E-04 | Male | 17.89% |
| 1522 | 0.000332573 | Male | 17.20% | 3.06E-04 | Male | 17.88% |
| 1521 | 0.000334095 | Male | 17.29% | 3.06E-04 | Male | 17.86% |
| 1520 | 0.00033218 | Male | 17.18% | 3.05E-04 | Male | 17.78% |
| 1519 | 0.000332037 | Male | 17.17% | 3.04E-04 | Male | 17.76% |
| 1518 | 0.000334251 | Male | 17.30% | 3.04E-04 | Male | 17.75% |
| 1517 | 0.00033065 | Male | 17.09% | 3.03E-04 | Male | 17.69% |
| 1516 | 0.000331098 | Male | 17.12% | 3.03E-04 | Male | 17.65% |
| 1515 | 0.000327774 | Male | 16.92% | 3.02E-04 | Male | 17.62% |
| 1514 | 0.000333402 | Male | 17.25% | 3.02E-04 | Male | 17.59% |
| 1513 | 0.000327956 | Male | 16.93% | 2.99E-04 | Male | 17.47% |
| 1512 | 0.000321733 | Male | 16.56% | 2.99E-04 | Male | 17.43% |
| 1511 | 0.000329508 | Male | 17.02% | 2.99E-04 | Male | 17.42% |
| 1510 | 0.000327937 | Male | 16.93% | 2.98E-04 | Male | 17.40% |
| 1509 | 0.000326993 | Male | 16.87% | 2.98E-04 | Male | 17.40% |
| 1508 | 0.000321757 | Male | 16.56% | 2.98E-04 | Male | 17.38% |
| 1507 | 0.000326615 | Male | 16.85% | 2.97E-04 | Male | 17.30% |
| 1506 | 0.000320673 | Male | 16.50% | 2.94E-04 | Male | 17.13% |
| 1505 | 0.000321735 | Male | 16.56% | 2.93E-04 | Male | 17.09% |
| 1504 | 0.000319813 | Male | 16.45% | 2.92E-04 | Male | 17.06% |
| 1503 | 0.000321517 | Male | 16.55% | 2.92E-04 | Male | 17.03% |
| 1502 | 0.000319541 | Male | 16.43% | 2.92E-04 | Male | 17.01% |
| 1501 | 0.000324412 | Male | 16.72% | 2.91E-04 | Male | 16.99% |
| 1500 | 0.000320461 | Male | 16.49% | 2.91E-04 | Male | 16.99% |
| 1499 | 0.000322214 | Male | 16.59% | 2.91E-04 | Male | 16.95% |
| 1498 | 0.000321883 | Male | 16.57% | 2.90E-04 | Male | 16.92% |
| 1497 | 0.000312184 | Male | 16.00% | 2.90E-04 | Male | 16.89% |
| 1496 | 0.000321472 | Male | 16.55% | 2.90E-04 | Male | 16.89% |
| 1495 | 0.000314179 | Male | 16.11% | 2.89E-04 | Male | 16.88% |
| 1494 | 0.000322308 | Male | 16.60% | 2.89E-04 | Male | 16.87% |
| 1493 | 0.000321136 | Male | 16.53% | 2.89E-04 | Male | 16.86% |
| 1492 | 0.000314071 | Male | 16.11% | 2.88E-04 | Male | 16.81% |
| 1491 | 0.000318722 | Male | 16.38% | 2.88E-04 | Male | 16.80% |
| 1490 | 0.000317528 | Male | 16.31% | 2.88E-04 | Male | 16.80% |
| 1489 | 0.000310831 | Male | 15.92% | 2.88E-04 | Male | 16.77% |
| 1488 | 0.00031549 | Male | 16.19% | 2.87E-04 | Male | 16.74% |
| 1487 | 0.000313906 | Male | 16.10% | 2.87E-04 | Male | 16.73% |
| 1486 | 0.000316355 | Male | 16.24% | 2.86E-04 | Male | 16.70% |
| 1485 | 0.000316079 | Male | 16.23% | 2.86E-04 | Male | 16.67% |
| 1484 | 0.0003118 | Male | 15.97% | 2.84E-04 | Male | 16.54% |
| 1483 | 0.000310089 | Male | 15.87% | 2.83E-04 | Male | 16.53% |
| 1482 | 0.000310231 | Male | 15.88% | 2.83E-04 | Male | 16.52% |
| 1481 | 0.000315811 | Male | 16.21% | 2.83E-04 | Male | 16.52% |
| 1480 | 0.000304825 | Male | 15.56% | 2.82E-04 | Male | 16.46% |
| 1479 | 0.000310492 | Male | 15.90% | 2.82E-04 | Male | 16.45% |
| 1478 | 0.000313098 | Male | 16.05% | 2.82E-04 | Male | 16.44% |
| 1477 | 0.000310524 | Male | 15.90% | 2.81E-04 | Male | 16.40% |
| 1476 | 0.000309145 | Male | 15.82% | 2.81E-04 | Male | 16.39% |
| 1475 | 0.000307495 | Male | 15.72% | 2.80E-04 | Male | 16.35% |
| 1474 | 0.000306249 | Male | 15.64% | 2.80E-04 | Male | 16.34% |
| 1473 | 0.000310893 | Male | 15.92% | 2.80E-04 | Male | 16.32% |
| 1472 | 0.000312505 | Male | 16.02% | 2.80E-04 | Male | 16.30% |
| 1471 | 0.000299627 | Male | 15.25% | 2.80E-04 | Male | 16.30% |
| 1470 | 0.000304797 | Male | 15.56% | 2.78E-04 | Male | 16.23% |
| 1469 | 0.00031284 | Male | 16.04% | 2.78E-04 | Male | 16.22% |
| 1468 | 0.000306386 | Male | 15.65% | 2.78E-04 | Male | 16.21% |
| 1467 | 0.000302831 | Male | 15.44% | 2.78E-04 | Male | 16.20% |
| 1466 | 0.000305096 | Male | 15.58% | 2.77E-04 | Male | 16.16% |
| 1465 | 0.000306814 | Male | 15.68% | 2.77E-04 | Male | 16.15% |
| 1464 | 0.000300614 | Male | 15.31% | 2.75E-04 | Male | 16.04% |
| 1463 | 0.00030314 | Male | 15.46% | 2.75E-04 | Male | 16.03% |
| 1462 | 0.000304367 | Male | 15.53% | 2.75E-04 | Male | 16.03% |
| 1461 | 0.000306436 | Male | 15.66% | 2.74E-04 | Male | 15.97% |
| 1460 | 0.000305135 | Male | 15.58% | 2.74E-04 | Male | 15.94% |
| 1459 | 0.000299749 | Male | 15.26% | 2.73E-04 | Male | 15.94% |
| 1458 | 0.000301807 | Male | 15.38% | 2.72E-04 | Male | 15.88% |
| 1457 | 0.000296916 | Male | 15.09% | 2.72E-04 | Male | 15.83% |
| 1456 | 0.00030179 | Male | 15.38% | 2.72E-04 | Male | 15.83% |
| 1455 | 0.000307331 | Male | 15.71% | 2.71E-04 | Male | 15.81% |
| 1454 | 0.000298414 | Male | 15.18% | 2.71E-04 | Male | 15.80% |
| 1453 | 0.000295484 | Male | 15.01% | 2.70E-04 | Male | 15.73% |
| 1452 | 0.000299449 | Male | 15.24% | 2.70E-04 | Male | 15.72% |
| 1451 | 0.000294731 | Male | 14.96% | 2.70E-04 | Male | 15.71% |
| 1450 | 0.000297503 | Male | 15.13% | 2.69E-04 | Male | 15.67% |
| 1449 | 0.000299035 | Male | 15.22% | 2.69E-04 | Male | 15.66% |
| 1448 | 0.000299745 | Male | 15.26% | 2.68E-04 | Male | 15.62% |
| 1447 | 0.000293287 | Male | 14.88% | 2.68E-04 | Male | 15.59% |
| 1446 | 0.000300142 | Male | 15.28% | 2.67E-04 | Male | 15.56% |
| 1445 | 0.000302266 | Male | 15.41% | 2.67E-04 | Male | 15.55% |
| 1444 | 0.00029806 | Male | 15.16% | 2.66E-04 | Male | 15.49% |
| 1443 | 0.000295029 | Male | 14.98% | 2.65E-04 | Male | 15.46% |
| 1442 | 0.000288986 | Male | 14.62% | 2.65E-04 | Male | 15.45% |
| 1441 | 0.000295473 | Male | 15.01% | 2.64E-04 | Male | 15.37% |
| 1440 | 0.000296327 | Male | 15.06% | 2.63E-04 | Male | 15.35% |
| 1439 | 0.000289371 | Male | 14.64% | 2.63E-04 | Male | 15.34% |
| 1438 | 0.000292097 | Male | 14.81% | 2.63E-04 | Male | 15.31% |
| 1437 | 0.000296089 | Male | 15.04% | 2.62E-04 | Male | 15.27% |
| 1436 | 0.00029277 | Male | 14.85% | 2.62E-04 | Male | 15.26% |
| 1435 | 0.000286267 | Male | 14.46% | 2.61E-04 | Male | 15.19% |
| 1434 | 0.000289711 | Male | 14.66% | 2.60E-04 | Male | 15.16% |
| 1433 | 0.000290963 | Male | 14.74% | 2.60E-04 | Male | 15.12% |
| 1432 | 0.000285963 | Male | 14.44% | 2.59E-04 | Male | 15.11% |
| 1431 | 0.000290012 | Male | 14.68% | 2.59E-04 | Male | 15.09% |
| 1430 | 0.000285482 | Male | 14.41% | 2.59E-04 | Male | 15.06% |
| 1429 | 0.000286024 | Male | 14.45% | 2.58E-04 | Male | 15.01% |
| 1428 | 0.000287041 | Male | 14.51% | 2.57E-04 | Male | 14.96% |
| 1427 | 0.000287561 | Male | 14.54% | 2.56E-04 | Male | 14.90% |
| 1426 | 0.000286854 | Male | 14.50% | 2.55E-04 | Male | 14.85% |
| 1425 | 0.000281554 | Male | 14.18% | 2.54E-04 | Male | 14.81% |
| 1424 | 0.000296497 | Male | 15.07% | 2.54E-04 | Male | 14.81% |
| 1423 | 0.000285042 | Male | 14.39% | 2.54E-04 | Male | 14.78% |
| 1422 | 0.000283484 | Male | 14.30% | 2.54E-04 | Male | 14.77% |
| 1421 | 0.000283994 | Male | 14.33% | 2.53E-04 | Male | 14.75% |
| 1420 | 0.000285325 | Male | 14.40% | 2.52E-04 | Male | 14.67% |
| 1419 | 0.00028557 | Male | 14.42% | 2.52E-04 | Male | 14.66% |
| 1418 | 0.000280176 | Male | 14.10% | 2.51E-04 | Male | 14.64% |
| 1417 | 0.000278721 | Male | 14.01% | 2.51E-04 | Male | 14.61% |
| 1416 | 0.000279829 | Male | 14.08% | 2.51E-04 | Male | 14.59% |
| 1415 | 0.000277218 | Male | 13.92% | 2.50E-04 | Male | 14.58% |
| 1414 | 0.000278023 | Male | 13.97% | 2.50E-04 | Male | 14.54% |
| 1413 | 0.000279063 | Male | 14.03% | 2.50E-04 | Male | 14.54% |
| 1412 | 0.000280803 | Male | 14.14% | 2.50E-04 | Male | 14.53% |
| 1411 | 0.000275714 | Male | 13.84% | 2.49E-04 | Male | 14.51% |
| 1410 | 0.000275794 | Male | 13.84% | 2.49E-04 | Male | 14.49% |
| 1409 | 0.000268836 | Male | 13.43% | 2.49E-04 | Male | 14.47% |
| 1408 | 0.000271891 | Male | 13.61% | 2.48E-04 | Male | 14.45% |
| 1407 | 0.000274922 | Male | 13.79% | 2.48E-04 | Male | 14.43% |
| 1406 | 0.000277388 | Male | 13.93% | 2.48E-04 | Male | 14.41% |
| 1405 | 0.000277651 | Male | 13.95% | 2.48E-04 | Male | 14.41% |
| 1404 | 0.000274571 | Male | 13.77% | 2.47E-04 | Male | 14.40% |
| 1403 | 0.000269702 | Male | 13.48% | 2.45E-04 | Male | 14.24% |
| 1402 | 0.000273032 | Male | 13.68% | 2.45E-04 | Male | 14.23% |
| 1401 | 0.000276136 | Male | 13.86% | 2.44E-04 | Male | 14.19% |
| 1400 | 0.000271994 | Male | 13.61% | 2.44E-04 | Male | 14.18% |
| 1399 | 0.000274201 | Male | 13.75% | 2.43E-04 | Male | 14.16% |
| 1398 | 0.000271813 | Male | 13.60% | 2.42E-04 | Male | 14.10% |
| 1397 | 0.000273232 | Male | 13.69% | 2.42E-04 | Male | 14.10% |
| 1396 | 0.00027039 | Male | 13.52% | 2.41E-04 | Male | 14.03% |
| 1395 | 0.000277629 | Male | 13.95% | 2.40E-04 | Male | 13.94% |
| 1394 | 0.0002658 | Male | 13.25% | 2.39E-04 | Male | 13.91% |
| 1393 | 0.00026268 | Male | 13.06% | 2.38E-04 | Male | 13.85% |
| 1392 | 0.000264548 | Male | 13.17% | 2.38E-04 | Male | 13.82% |
| 1391 | 0.000265578 | Male | 13.23% | 2.37E-04 | Male | 13.77% |
| 1390 | 0.000270555 | Male | 13.53% | 2.36E-04 | Male | 13.75% |
| 1389 | 0.000261399 | Male | 12.99% | 2.36E-04 | Male | 13.72% |
| 1388 | 0.000263756 | Male | 13.13% | 2.35E-04 | Male | 13.69% |
| 1387 | 0.000265826 | Male | 13.25% | 2.35E-04 | Male | 13.69% |
| 1386 | 0.000264239 | Male | 13.16% | 2.35E-04 | Male | 13.68% |
| 1385 | 0.000265547 | Male | 13.23% | 2.35E-04 | Male | 13.65% |
| 1384 | 0.000260183 | Male | 12.92% | 2.35E-04 | Male | 13.64% |
| 1383 | 0.00026602 | Male | 13.26% | 2.34E-04 | Male | 13.63% |
| 1382 | 0.000260946 | Male | 12.96% | 2.34E-04 | Male | 13.61% |
| 1381 | 0.000255615 | Male | 12.64% | 2.34E-04 | Male | 13.61% |
| 1380 | 0.000265065 | Male | 13.20% | 2.34E-04 | Male | 13.60% |
| 1379 | 0.000258445 | Male | 12.81% | 2.34E-04 | Male | 13.59% |
| 1378 | 0.000263733 | Male | 13.13% | 2.33E-04 | Male | 13.57% |
| 1377 | 0.000266347 | Male | 13.28% | 2.33E-04 | Male | 13.55% |
| 1376 | 0.000261499 | Male | 12.99% | 2.33E-04 | Male | 13.55% |
| 1375 | 0.000268254 | Male | 13.39% | 2.33E-04 | Male | 13.54% |
| 1374 | 0.000257635 | Male | 12.76% | 2.32E-04 | Male | 13.50% |
| 1373 | 0.00026713 | Male | 13.33% | 2.32E-04 | Male | 13.49% |
| 1372 | 0.000255885 | Male | 12.66% | 2.32E-04 | Male | 13.49% |
| 1371 | 0.000264265 | Male | 13.16% | 2.32E-04 | Male | 13.49% |
| 1370 | 0.0002572 | Male | 12.74% | 2.32E-04 | Male | 13.47% |
| 1369 | 0.000261553 | Male | 13.00% | 2.31E-04 | Male | 13.46% |
| 1368 | 0.000253689 | Male | 12.53% | 2.31E-04 | Male | 13.45% |
| 1367 | 0.000261106 | Male | 12.97% | 2.31E-04 | Male | 13.45% |
| 1366 | 0.000257121 | Male | 12.73% | 2.31E-04 | Male | 13.44% |
| 1365 | 0.000259009 | Male | 12.85% | 2.31E-04 | Male | 13.43% |
| 1364 | 0.000260094 | Male | 12.91% | 2.31E-04 | Male | 13.42% |
| 1363 | 0.000257351 | Male | 12.75% | 2.30E-04 | Male | 13.38% |
| 1362 | 0.000256387 | Male | 12.69% | 2.30E-04 | Male | 13.37% |
| 1361 | 0.000254526 | Male | 12.58% | 2.29E-04 | Male | 13.34% |
| 1360 | 0.000254817 | Male | 12.60% | 2.29E-04 | Male | 13.29% |
| 1359 | 0.000257319 | Male | 12.75% | 2.28E-04 | Male | 13.28% |
| 1358 | 0.000257425 | Male | 12.75% | 2.28E-04 | Male | 13.27% |
| 1357 | 0.000254396 | Male | 12.57% | 2.28E-04 | Male | 13.26% |
| 1356 | 0.000260168 | Male | 12.91% | 2.28E-04 | Male | 13.25% |
| 1355 | 0.000261611 | Male | 13.00% | 2.28E-04 | Male | 13.24% |
| 1354 | 0.00025803 | Male | 12.79% | 2.27E-04 | Male | 13.22% |
| 1353 | 0.000256029 | Male | 12.67% | 2.27E-04 | Male | 13.22% |
| 1352 | 0.000264655 | Male | 13.18% | 2.27E-04 | Male | 13.19% |
| 1351 | 0.000257992 | Male | 12.79% | 2.27E-04 | Male | 13.18% |
| 1350 | 0.000254214 | Male | 12.56% | 2.27E-04 | Male | 13.18% |
| 1349 | 0.000253082 | Male | 12.49% | 2.27E-04 | Male | 13.17% |
| 1348 | 0.000255579 | Male | 12.64% | 2.26E-04 | Male | 13.17% |
| 1347 | 0.000254906 | Male | 12.60% | 2.26E-04 | Male | 13.15% |
| 1346 | 0.000251742 | Male | 12.42% | 2.26E-04 | Male | 13.12% |
| 1345 | 0.000251218 | Male | 12.38% | 2.26E-04 | Male | 13.12% |
| 1344 | 0.000254069 | Male | 12.55% | 2.25E-04 | Male | 13.11% |
| 1343 | 0.000250572 | Male | 12.35% | 2.25E-04 | Male | 13.06% |
| 1342 | 0.000252528 | Male | 12.46% | 2.24E-04 | Male | 13.05% |
| 1341 | 0.000249146 | Male | 12.26% | 2.24E-04 | Male | 13.02% |
| 1340 | 0.000252565 | Male | 12.46% | 2.23E-04 | Male | 12.99% |
| 1339 | 0.00025207 | Male | 12.43% | 2.23E-04 | Male | 12.99% |
| 1338 | 0.000253951 | Male | 12.55% | 2.23E-04 | Male | 12.97% |
| 1337 | 0.000249489 | Male | 12.28% | 2.23E-04 | Male | 12.95% |
| 1336 | 0.000255451 | Male | 12.63% | 2.22E-04 | Male | 12.93% |
| 1335 | 0.000252585 | Male | 12.47% | 2.22E-04 | Male | 12.90% |
| 1334 | 0.000251449 | Male | 12.40% | 2.21E-04 | Male | 12.85% |
| 1333 | 0.000247439 | Male | 12.16% | 2.21E-04 | Male | 12.83% |
| 1332 | 0.000253145 | Male | 12.50% | 2.21E-04 | Male | 12.83% |
| 1331 | 0.000246793 | Male | 12.12% | 2.20E-04 | Male | 12.81% |
| 1330 | 0.000246421 | Male | 12.10% | 2.20E-04 | Male | 12.79% |
| 1329 | 0.000247855 | Male | 12.18% | 2.20E-04 | Male | 12.76% |
| 1328 | 0.000251722 | Male | 12.41% | 2.20E-04 | Male | 12.76% |
| 1327 | 0.000244706 | Male | 12.00% | 2.19E-04 | Male | 12.74% |
| 1326 | 0.000244527 | Male | 11.99% | 2.19E-04 | Male | 12.73% |
| 1325 | 0.000244532 | Male | 11.99% | 2.19E-04 | Male | 12.71% |
| 1324 | 0.000253726 | Male | 12.53% | 2.18E-04 | Male | 12.70% |
| 1323 | 0.000245954 | Male | 12.07% | 2.18E-04 | Male | 12.69% |
| 1322 | 0.000249744 | Male | 12.30% | 2.18E-04 | Male | 12.69% |
| 1321 | 0.000246307 | Male | 12.09% | 2.18E-04 | Male | 12.66% |
| 1320 | 0.000246072 | Male | 12.08% | 2.17E-04 | Male | 12.63% |
| 1319 | 0.00024123 | Male | 11.79% | 2.16E-04 | Male | 12.57% |
| 1318 | 0.000241373 | Male | 11.80% | 2.16E-04 | Male | 12.56% |
| 1317 | 0.000245943 | Male | 12.07% | 2.16E-04 | Male | 12.56% |
| 1316 | 0.00024252 | Male | 11.87% | 2.16E-04 | Male | 12.55% |
| 1315 | 0.000241152 | Male | 11.79% | 2.16E-04 | Male | 12.55% |
| 1314 | 0.000243811 | Male | 11.95% | 2.16E-04 | Male | 12.54% |
| 1313 | 0.000244135 | Male | 11.96% | 2.16E-04 | Male | 12.54% |
| 1312 | 0.000244788 | Male | 12.00% | 2.15E-04 | Male | 12.48% |
| 1311 | 0.000242762 | Male | 11.88% | 2.15E-04 | Male | 12.47% |
| 1310 | 0.000243899 | Male | 11.95% | 2.14E-04 | Male | 12.44% |
| 1309 | 0.000245149 | Male | 12.02% | 2.14E-04 | Male | 12.43% |
| 1308 | 0.000238669 | Male | 11.64% | 2.14E-04 | Male | 12.41% |
| 1307 | 0.000238573 | Male | 11.63% | 2.13E-04 | Male | 12.39% |
| 1306 | 0.000238022 | Male | 11.60% | 2.13E-04 | Male | 12.38% |
| 1305 | 0.000242476 | Male | 11.87% | 2.13E-04 | Male | 12.37% |
| 1304 | 0.000240085 | Male | 11.72% | 2.13E-04 | Male | 12.37% |
| 1303 | 0.000242398 | Male | 11.86% | 2.13E-04 | Male | 12.36% |
| 1302 | 0.000238077 | Male | 11.61% | 2.13E-04 | Male | 12.35% |
| 1301 | 0.000236273 | Male | 11.50% | 2.12E-04 | Male | 12.34% |
| 1300 | 0.000241484 | Male | 11.81% | 2.11E-04 | Male | 12.28% |
| 1299 | 0.000233253 | Male | 11.32% | 2.11E-04 | Male | 12.27% |
| 1298 | 0.000243904 | Male | 11.95% | 2.11E-04 | Male | 12.27% |
| 1297 | 0.000241494 | Male | 11.81% | 2.11E-04 | Male | 12.26% |
| 1296 | 0.000239096 | Male | 11.67% | 2.11E-04 | Male | 12.26% |
| 1295 | 0.000240326 | Male | 11.74% | 2.11E-04 | Male | 12.25% |
| 1294 | 0.000244343 | Male | 11.98% | 2.11E-04 | Male | 12.25% |
| 1293 | 0.000241687 | Male | 11.82% | 2.10E-04 | Male | 12.22% |
| 1292 | 0.000240396 | Male | 11.74% | 2.10E-04 | Male | 12.21% |
| 1291 | 0.00023689 | Male | 11.54% | 2.10E-04 | Male | 12.20% |
| 1290 | 0.000232425 | Male | 11.27% | 2.10E-04 | Male | 12.19% |
| 1289 | 0.00023808 | Male | 11.61% | 2.09E-04 | Male | 12.14% |
| 1288 | 0.000235922 | Male | 11.48% | 2.09E-04 | Male | 12.14% |
| 1287 | 0.00024056 | Male | 11.75% | 2.09E-04 | Male | 12.14% |
| 1286 | 0.000240403 | Male | 11.74% | 2.09E-04 | Male | 12.12% |
| 1285 | 0.000235895 | Male | 11.48% | 2.08E-04 | Male | 12.09% |
| 1284 | 0.000232734 | Male | 11.29% | 2.08E-04 | Male | 12.06% |
| 1283 | 0.000232897 | Male | 11.30% | 2.07E-04 | Male | 12.05% |
| 1282 | 0.000235059 | Male | 11.43% | 2.07E-04 | Male | 12.01% |
| 1281 | 0.00023967 | Male | 11.70% | 2.07E-04 | Male | 12.00% |
| 1280 | 0.000234007 | Male | 11.36% | 2.06E-04 | Male | 11.97% |
| 1279 | 0.000238231 | Male | 11.61% | 2.06E-04 | Male | 11.96% |
| 1278 | 0.000238552 | Male | 11.63% | 2.06E-04 | Male | 11.96% |
| 1277 | 0.000235017 | Male | 11.42% | 2.06E-04 | Male | 11.96% |
| 1276 | 0.000230425 | Male | 11.15% | 2.05E-04 | Male | 11.93% |
| 1275 | 0.000230333 | Male | 11.15% | 2.05E-04 | Male | 11.92% |
| 1274 | 0.00022762 | Male | 10.99% | 2.05E-04 | Male | 11.90% |
| 1273 | 0.000229084 | Male | 11.07% | 2.05E-04 | Male | 11.88% |
| 1272 | 0.000237847 | Male | 11.59% | 2.05E-04 | Male | 11.88% |
| 1271 | 0.000235724 | Male | 11.47% | 2.04E-04 | Male | 11.86% |
| 1270 | 0.000231963 | Male | 11.24% | 2.04E-04 | Male | 11.83% |
| 1269 | 0.000228733 | Male | 11.05% | 2.04E-04 | Male | 11.82% |
| 1268 | 0.000237659 | Male | 11.58% | 2.03E-04 | Male | 11.80% |
| 1267 | 0.000233789 | Male | 11.35% | 2.03E-04 | Male | 11.79% |
| 1266 | 0.000234105 | Male | 11.37% | 2.03E-04 | Male | 11.79% |
| 1265 | 0.00023098 | Male | 11.18% | 2.03E-04 | Male | 11.78% |
| 1264 | 0.00023399 | Male | 11.36% | 2.03E-04 | Male | 11.77% |
| 1263 | 0.000228344 | Male | 11.03% | 2.01E-04 | Male | 11.65% |
| 1262 | 0.000229963 | Male | 11.12% | 2.01E-04 | Male | 11.64% |
| 1261 | 0.000226742 | Male | 10.93% | 2.00E-04 | Male | 11.63% |
| 1260 | 0.00022966 | Male | 11.11% | 2.00E-04 | Male | 11.61% |
| 1259 | 0.000231434 | Male | 11.21% | 2.00E-04 | Male | 11.60% |
| 1258 | 0.000222917 | Male | 10.71% | 2.00E-04 | Male | 11.59% |
| 1257 | 0.000221715 | Male | 10.64% | 1.99E-04 | Male | 11.57% |
| 1256 | 0.000226516 | Male | 10.92% | 1.99E-04 | Male | 11.56% |
| 1255 | 0.000226739 | Male | 10.93% | 1.99E-04 | Male | 11.55% |
| 1254 | 0.000226199 | Male | 10.90% | 1.99E-04 | Male | 11.55% |
| 1253 | 0.000227377 | Male | 10.97% | 1.99E-04 | Male | 11.53% |
| 1252 | 0.000226127 | Male | 10.90% | 1.98E-04 | Male | 11.52% |
| 1251 | 0.000225655 | Male | 10.87% | 1.98E-04 | Male | 11.50% |
| 1250 | 0.000223015 | Male | 10.71% | 1.98E-04 | Male | 11.49% |
| 1249 | 0.000221991 | Male | 10.65% | 1.98E-04 | Male | 11.47% |
| 1248 | 0.000226201 | Male | 10.90% | 1.97E-04 | Male | 11.45% |
| 1247 | 0.000223139 | Male | 10.72% | 1.97E-04 | Male | 11.45% |
| 1246 | 0.000230048 | Male | 11.13% | 1.97E-04 | Male | 11.44% |
| 1245 | 0.000222543 | Male | 10.69% | 1.96E-04 | Male | 11.39% |
| 1244 | 0.000223768 | Male | 10.76% | 1.96E-04 | Male | 11.36% |
| 1243 | 0.000218708 | Male | 10.46% | 1.96E-04 | Male | 11.36% |
| 1242 | 0.000225752 | Male | 10.88% | 1.95E-04 | Male | 11.33% |
| 1241 | 0.00022208 | Male | 10.66% | 1.95E-04 | Male | 11.28% |
| 1240 | 0.000223711 | Male | 10.75% | 1.94E-04 | Male | 11.28% |
| 1239 | 0.000218809 | Male | 10.46% | 1.94E-04 | Male | 11.28% |
| 1238 | 0.000225109 | Male | 10.84% | 1.94E-04 | Male | 11.27% |
| 1237 | 0.000222923 | Male | 10.71% | 1.93E-04 | Male | 11.20% |
| 1236 | 0.000220457 | Male | 10.56% | 1.93E-04 | Male | 11.18% |
| 1235 | 0.000223435 | Male | 10.74% | 1.93E-04 | Male | 11.18% |
| 1234 | 0.000219249 | Male | 10.49% | 1.92E-04 | Male | 11.14% |
| 1233 | 0.000222996 | Male | 10.71% | 1.92E-04 | Male | 11.12% |
| 1232 | 0.000220825 | Male | 10.58% | 1.92E-04 | Male | 11.11% |
| 1231 | 0.0002224 | Male | 10.68% | 1.92E-04 | Male | 11.11% |
| 1230 | 0.000219765 | Male | 10.52% | 1.91E-04 | Male | 11.10% |
| 1229 | 0.000215186 | Male | 10.25% | 1.91E-04 | Male | 11.10% |
| 1228 | 0.000217536 | Male | 10.39% | 1.91E-04 | Male | 11.08% |
| 1227 | 0.000214258 | Male | 10.19% | 1.91E-04 | Male | 11.07% |
| 1226 | 0.000219958 | Male | 10.53% | 1.91E-04 | Male | 11.07% |
| 1225 | 0.000217371 | Male | 10.38% | 1.90E-04 | Male | 11.02% |
| 1224 | 0.00021515 | Male | 10.25% | 1.90E-04 | Male | 11.00% |
| 1223 | 0.000219224 | Male | 10.49% | 1.90E-04 | Male | 10.99% |
| 1222 | 0.000215424 | Male | 10.26% | 1.89E-04 | Male | 10.98% |
| 1221 | 0.000216699 | Male | 10.34% | 1.89E-04 | Male | 10.98% |
| 1220 | 0.000215781 | Male | 10.28% | 1.89E-04 | Male | 10.97% |
| 1219 | 0.000217654 | Male | 10.40% | 1.89E-04 | Male | 10.96% |
| 1218 | 0.000218083 | Male | 10.42% | 1.89E-04 | Male | 10.95% |
| 1217 | 0.000223284 | Male | 10.73% | 1.89E-04 | Male | 10.95% |
| 1216 | 0.000215483 | Male | 10.27% | 1.89E-04 | Male | 10.94% |
| 1215 | 0.000213229 | Male | 10.13% | 1.88E-04 | Male | 10.92% |
| 1214 | 0.000214902 | Male | 10.23% | 1.88E-04 | Male | 10.91% |
| 1213 | 0.000215385 | Male | 10.26% | 1.88E-04 | Male | 10.88% |
| 1212 | 0.000218314 | Male | 10.43% | 1.88E-04 | Male | 10.88% |
| 1211 | 0.000210628 | Male | 9.98% | 1.87E-04 | Male | 10.86% |
| 1210 | 0.000213531 | Male | 10.15% | 1.87E-04 | Male | 10.86% |
| 1209 | 0.000214582 | Male | 10.21% | 1.87E-04 | Male | 10.86% |
| 1208 | 0.000217526 | Male | 10.39% | 1.87E-04 | Male | 10.85% |
| 1207 | 0.000210679 | Male | 9.98% | 1.87E-04 | Male | 10.85% |
| 1206 | 0.000216034 | Male | 10.30% | 1.87E-04 | Male | 10.84% |
| 1205 | 0.000218594 | Male | 10.45% | 1.87E-04 | Male | 10.83% |
| 1204 | 0.000214267 | Male | 10.19% | 1.87E-04 | Male | 10.83% |
| 1203 | 0.000212413 | Male | 10.08% | 1.87E-04 | Male | 10.82% |
| 1202 | 0.000210095 | Male | 9.95% | 1.86E-04 | Male | 10.78% |
| 1201 | 0.00021598 | Male | 10.30% | 1.86E-04 | Male | 10.78% |
| 1200 | 0.000208174 | Male | 9.83% | 1.86E-04 | Male | 10.77% |
| 1199 | 0.000212549 | Male | 10.09% | 1.86E-04 | Male | 10.77% |
| 1198 | 0.000205484 | Male | 9.67% | 1.86E-04 | Male | 10.76% |
| 1197 | 0.00021432 | Male | 10.20% | 1.85E-04 | Male | 10.75% |
| 1196 | 0.000217798 | Male | 10.40% | 1.85E-04 | Male | 10.71% |
| 1195 | 0.000212715 | Male | 10.10% | 1.85E-04 | Male | 10.70% |
| 1194 | 0.000208407 | Male | 9.85% | 1.84E-04 | Male | 10.67% |
| 1193 | 0.0002155 | Male | 10.27% | 1.84E-04 | Male | 10.66% |
| 1192 | 0.000212684 | Male | 10.10% | 1.84E-04 | Male | 10.66% |
| 1191 | 0.000209581 | Male | 9.92% | 1.84E-04 | Male | 10.64% |
| 1190 | 0.00021304 | Male | 10.12% | 1.83E-04 | Male | 10.61% |
| 1189 | 0.000211637 | Male | 10.04% | 1.83E-04 | Male | 10.60% |
| 1188 | 0.000210866 | Male | 9.99% | 1.83E-04 | Male | 10.60% |
| 1187 | 0.000211008 | Male | 10.00% | 1.83E-04 | Male | 10.59% |
| 1186 | 0.000208133 | Male | 9.83% | 1.82E-04 | Male | 10.57% |
| 1185 | 0.000208131 | Male | 9.83% | 1.82E-04 | Male | 10.56% |
| 1184 | 0.000205658 | Male | 9.68% | 1.82E-04 | Male | 10.56% |
| 1183 | 0.000213418 | Male | 10.14% | 1.82E-04 | Male | 10.56% |
| 1182 | 0.000210296 | Male | 9.96% | 1.82E-04 | Male | 10.56% |
| 1181 | 0.000210401 | Male | 9.97% | 1.82E-04 | Male | 10.55% |
| 1180 | 0.000209589 | Male | 9.92% | 1.82E-04 | Male | 10.54% |
| 1179 | 0.000212082 | Male | 10.07% | 1.82E-04 | Male | 10.54% |
| 1178 | 0.000210322 | Male | 9.96% | 1.82E-04 | Male | 10.54% |
| 1177 | 0.000212603 | Male | 10.10% | 1.82E-04 | Male | 10.52% |
| 1176 | 0.000208225 | Male | 9.84% | 1.81E-04 | Male | 10.51% |
| 1175 | 0.00020582 | Male | 9.69% | 1.81E-04 | Male | 10.50% |
| 1174 | 0.000207572 | Male | 9.80% | 1.81E-04 | Male | 10.50% |
| 1173 | 0.00020022 | Male | 9.36% | 1.80E-04 | Male | 10.45% |
| 1172 | 0.000205727 | Male | 9.69% | 1.80E-04 | Male | 10.43% |
| 1171 | 0.000211459 | Male | 10.03% | 1.80E-04 | Male | 10.40% |
| 1170 | 0.000203225 | Male | 9.54% | 1.80E-04 | Male | 10.40% |
| 1169 | 0.00020527 | Male | 9.66% | 1.79E-04 | Male | 10.39% |
| 1168 | 0.000207615 | Male | 9.80% | 1.79E-04 | Male | 10.39% |
| 1167 | 0.000203504 | Male | 9.56% | 1.79E-04 | Male | 10.39% |
| 1166 | 0.000208086 | Male | 9.83% | 1.79E-04 | Male | 10.38% |
| 1165 | 0.000207694 | Male | 9.81% | 1.79E-04 | Male | 10.34% |
| 1164 | 0.000210325 | Male | 9.96% | 1.79E-04 | Male | 10.34% |
| 1163 | 0.000204603 | Male | 9.62% | 1.79E-04 | Male | 10.34% |
| 1162 | 0.000202481 | Male | 9.50% | 1.78E-04 | Male | 10.34% |
| 1161 | 0.000203471 | Male | 9.56% | 1.78E-04 | Male | 10.32% |
| 1160 | 0.000204244 | Male | 9.60% | 1.78E-04 | Male | 10.31% |
| 1159 | 0.000206718 | Male | 9.75% | 1.78E-04 | Male | 10.31% |
| 1158 | 0.000208543 | Male | 9.86% | 1.78E-04 | Male | 10.30% |
| 1157 | 0.000208508 | Male | 9.85% | 1.78E-04 | Male | 10.30% |
| 1156 | 0.000205047 | Male | 9.65% | 1.77E-04 | Male | 10.27% |
| 1155 | 0.00020211 | Male | 9.47% | 1.77E-04 | Male | 10.26% |
| 1154 | 0.000201969 | Male | 9.47% | 1.77E-04 | Male | 10.22% |
| 1153 | 0.000200872 | Male | 9.40% | 1.76E-04 | Male | 10.22% |
| 1152 | 0.000206052 | Male | 9.71% | 1.76E-04 | Male | 10.21% |
| 1151 | 0.000195521 | Male | 9.08% | 1.76E-04 | Male | 10.20% |
| 1150 | 0.000197125 | Male | 9.18% | 1.76E-04 | Male | 10.18% |
| 1149 | 0.00020267 | Male | 9.51% | 1.76E-04 | Male | 10.16% |
| 1148 | 0.000207059 | Male | 9.77% | 1.75E-04 | Male | 10.16% |
| 1147 | 0.000208705 | Male | 9.87% | 1.75E-04 | Male | 10.15% |
| 1146 | 0.000206132 | Male | 9.71% | 1.75E-04 | Male | 10.14% |
| 1145 | 0.000197317 | Male | 9.19% | 1.75E-04 | Male | 10.13% |
| 1144 | 0.000196119 | Male | 9.12% | 1.74E-04 | Male | 10.10% |
| 1143 | 0.000205476 | Male | 9.67% | 1.74E-04 | Male | 10.09% |
| 1142 | 0.000205849 | Male | 9.70% | 1.74E-04 | Male | 10.08% |
| 1141 | 0.000205133 | Male | 9.65% | 1.74E-04 | Male | 10.07% |
| 1140 | 0.000200922 | Male | 9.40% | 1.73E-04 | Male | 10.04% |
| 1139 | 0.000195303 | Male | 9.07% | 1.73E-04 | Male | 10.03% |
| 1138 | 0.000197263 | Male | 9.19% | 1.73E-04 | Male | 10.02% |
| 1137 | 0.000202885 | Male | 9.52% | 1.73E-04 | Male | 10.00% |
| 1136 | 0.000200269 | Male | 9.37% | 1.72E-04 | Male | 9.98% |
| 1135 | 0.000196234 | Male | 9.13% | 1.72E-04 | Male | 9.98% |
| 1134 | 0.000198362 | Male | 9.25% | 1.72E-04 | Male | 9.95% |
| 1133 | 0.00020276 | Male | 9.51% | 1.72E-04 | Male | 9.94% |
| 1132 | 0.000195412 | Male | 9.08% | 1.72E-04 | Male | 9.94% |
| 1131 | 0.00020238 | Male | 9.49% | 1.72E-04 | Male | 9.94% |
| 1130 | 0.000208773 | Male | 9.87% | 1.72E-04 | Male | 9.94% |
| 1129 | 0.000196983 | Male | 9.17% | 1.72E-04 | Male | 9.93% |
| 1128 | 0.000199389 | Male | 9.31% | 1.71E-04 | Male | 9.91% |
| 1127 | 0.000195787 | Male | 9.10% | 1.71E-04 | Male | 9.88% |
| 1126 | 0.000195998 | Male | 9.11% | 1.70E-04 | Male | 9.85% |
| 1125 | 0.000196878 | Male | 9.16% | 1.70E-04 | Male | 9.82% |
| 1124 | 0.000193984 | Male | 8.99% | 1.69E-04 | Male | 9.81% |
| 1123 | 0.000198736 | Male | 9.27% | 1.69E-04 | Male | 9.77% |
| 1122 | 0.000195833 | Male | 9.10% | 1.69E-04 | Male | 9.76% |
| 1121 | 0.000191815 | Male | 8.86% | 1.69E-04 | Male | 9.75% |
| 1120 | 0.000192999 | Male | 8.93% | 1.69E-04 | Male | 9.75% |
| 1119 | 0.000194066 | Male | 9.00% | 1.68E-04 | Male | 9.75% |
| 1118 | 0.000195296 | Male | 9.07% | 1.68E-04 | Male | 9.72% |
| 1117 | 0.000195039 | Male | 9.06% | 1.68E-04 | Male | 9.71% |
| 1116 | 0.000194831 | Male | 9.04% | 1.68E-04 | Male | 9.70% |
| 1115 | 0.000199336 | Male | 9.31% | 1.67E-04 | Male | 9.69% |
| 1114 | 0.00019713 | Male | 9.18% | 1.67E-04 | Male | 9.67% |
| 1113 | 0.000191165 | Male | 8.83% | 1.66E-04 | Male | 9.63% |
| 1112 | 0.000194977 | Male | 9.05% | 1.66E-04 | Male | 9.62% |
| 1111 | 0.000200319 | Male | 9.37% | 1.66E-04 | Male | 9.59% |
| 1110 | 0.000186805 | Male | 8.57% | 1.66E-04 | Male | 9.59% |
| 1109 | 0.000191551 | Male | 8.85% | 1.65E-04 | Male | 9.54% |
| 1108 | 0.000186171 | Male | 8.53% | 1.65E-04 | Male | 9.53% |
| 1107 | 0.000190667 | Male | 8.80% | 1.64E-04 | Male | 9.50% |
| 1106 | 0.000191435 | Male | 8.84% | 1.64E-04 | Male | 9.50% |
| 1105 | 0.000190914 | Male | 8.81% | 1.64E-04 | Male | 9.50% |
| 1104 | 0.000191361 | Male | 8.84% | 1.64E-04 | Male | 9.49% |
| 1103 | 0.000191485 | Male | 8.84% | 1.64E-04 | Male | 9.49% |
| 1102 | 0.000188821 | Male | 8.69% | 1.63E-04 | Male | 9.44% |
| 1101 | 0.000193571 | Male | 8.97% | 1.63E-04 | Male | 9.42% |
| 1100 | 0.000193946 | Male | 8.99% | 1.63E-04 | Male | 9.42% |
| 1099 | 0.000188241 | Male | 8.65% | 1.63E-04 | Male | 9.41% |
| 1098 | 0.000186592 | Male | 8.56% | 1.63E-04 | Male | 9.40% |
| 1097 | 0.000189052 | Male | 8.70% | 1.62E-04 | Male | 9.40% |
| 1096 | 0.000186911 | Male | 8.57% | 1.62E-04 | Male | 9.40% |
| 1095 | 0.000186884 | Male | 8.57% | 1.62E-04 | Male | 9.39% |
| 1094 | 0.000185191 | Male | 8.47% | 1.62E-04 | Male | 9.37% |
| 1093 | 0.000190385 | Male | 8.78% | 1.62E-04 | Male | 9.37% |
| 1092 | 0.000191746 | Male | 8.86% | 1.62E-04 | Male | 9.37% |
| 1091 | 0.000185703 | Male | 8.50% | 1.62E-04 | Male | 9.36% |
| 1090 | 0.000189226 | Male | 8.71% | 1.62E-04 | Male | 9.36% |
| 1089 | 0.000191153 | Male | 8.83% | 1.62E-04 | Male | 9.36% |
| 1088 | 0.000186376 | Male | 8.54% | 1.62E-04 | Male | 9.35% |
| 1087 | 0.000189528 | Male | 8.73% | 1.62E-04 | Male | 9.34% |
| 1086 | 0.000186863 | Male | 8.57% | 1.61E-04 | Male | 9.33% |
| 1085 | 0.000187886 | Male | 8.63% | 1.61E-04 | Male | 9.30% |
| 1084 | 0.000190643 | Male | 8.80% | 1.61E-04 | Male | 9.30% |
| 1083 | 0.000191158 | Male | 8.83% | 1.61E-04 | Male | 9.28% |
| 1082 | 0.000189482 | Male | 8.73% | 1.60E-04 | Male | 9.24% |
| 1081 | 0.000184582 | Male | 8.44% | 1.60E-04 | Male | 9.24% |
| 1080 | 0.000184346 | Male | 8.42% | 1.60E-04 | Male | 9.24% |
| 1079 | 0.000190483 | Male | 8.79% | 1.59E-04 | Male | 9.21% |
| 1078 | 0.000191087 | Male | 8.82% | 1.59E-04 | Male | 9.21% |
| 1077 | 0.000185073 | Male | 8.47% | 1.59E-04 | Male | 9.20% |
| 1076 | 0.000186408 | Male | 8.54% | 1.59E-04 | Male | 9.19% |
| 1075 | 0.000185969 | Male | 8.52% | 1.59E-04 | Male | 9.18% |
| 1074 | 0.000184859 | Male | 8.45% | 1.59E-04 | Male | 9.17% |
| 1073 | 0.000183593 | Male | 8.38% | 1.59E-04 | Male | 9.16% |
| 1072 | 0.000190315 | Male | 8.78% | 1.58E-04 | Male | 9.14% |
| 1071 | 0.000187493 | Male | 8.61% | 1.58E-04 | Male | 9.13% |
| 1070 | 0.000188865 | Male | 8.69% | 1.58E-04 | Male | 9.12% |
| 1069 | 0.000183808 | Male | 8.39% | 1.58E-04 | Male | 9.12% |
| 1068 | 0.000189548 | Male | 8.73% | 1.57E-04 | Male | 9.09% |
| 1067 | 0.000181588 | Male | 8.26% | 1.57E-04 | Male | 9.05% |
| 1066 | 0.00018287 | Male | 8.33% | 1.56E-04 | Male | 9.04% |
| 1065 | 0.000187827 | Male | 8.63% | 1.56E-04 | Male | 9.03% |
| 1064 | 0.000181927 | Male | 8.28% | 1.56E-04 | Male | 9.03% |
| 1063 | 0.000177272 | Male | 8.00% | 1.56E-04 | Male | 9.02% |
| 1062 | 0.000179905 | Male | 8.16% | 1.56E-04 | Male | 9.01% |
| 1061 | 0.000180553 | Male | 8.20% | 1.56E-04 | Male | 8.99% |
| 1060 | 0.000181587 | Male | 8.26% | 1.55E-04 | Male | 8.98% |
| 1059 | 0.000185175 | Male | 8.47% | 1.55E-04 | Male | 8.96% |
| 1058 | 0.000178504 | Male | 8.08% | 1.55E-04 | Male | 8.95% |
| 1057 | 0.000179112 | Male | 8.11% | 1.55E-04 | Male | 8.94% |
| 1056 | 0.000180201 | Male | 8.18% | 1.54E-04 | Male | 8.92% |
| 1055 | 0.000177936 | Male | 8.04% | 1.54E-04 | Male | 8.90% |
| 1054 | 0.000180681 | Male | 8.20% | 1.54E-04 | Male | 8.90% |
| 1053 | 0.000184283 | Male | 8.42% | 1.54E-04 | Male | 8.88% |
| 1052 | 0.00018425 | Male | 8.42% | 1.53E-04 | Male | 8.84% |
| 1051 | 0.000185025 | Male | 8.46% | 1.53E-04 | Male | 8.83% |
| 1050 | 0.000178225 | Male | 8.06% | 1.53E-04 | Male | 8.83% |
| 1049 | 0.000178444 | Male | 8.07% | 1.53E-04 | Male | 8.82% |
| 1048 | 0.000181726 | Male | 8.27% | 1.52E-04 | Male | 8.80% |
| 1047 | 0.000179317 | Male | 8.12% | 1.52E-04 | Male | 8.77% |
| 1046 | 0.00018131 | Male | 8.24% | 1.52E-04 | Male | 8.77% |
| 1045 | 0.000180637 | Male | 8.20% | 1.51E-04 | Male | 8.75% |
| 1044 | 0.000178988 | Male | 8.10% | 1.51E-04 | Male | 8.75% |
| 1043 | 0.000179557 | Male | 8.14% | 1.51E-04 | Male | 8.74% |
| 1042 | 0.000181352 | Male | 8.24% | 1.51E-04 | Male | 8.73% |
| 1041 | 0.000178393 | Male | 8.07% | 1.51E-04 | Male | 8.73% |
| 1040 | 0.000178716 | Male | 8.09% | 1.50E-04 | Male | 8.69% |
| 1039 | 0.00017452 | Male | 7.84% | 1.50E-04 | Male | 8.67% |
| 1038 | 0.00017756 | Male | 8.02% | 1.50E-04 | Male | 8.67% |
| 1037 | 0.0001724 | Male | 7.71% | 1.50E-04 | Male | 8.65% |
| 1036 | 0.000172216 | Male | 7.70% | 1.48E-04 | Male | 8.57% |
| 1035 | 0.000177444 | Male | 8.01% | 1.48E-04 | Male | 8.54% |
| 1034 | 0.000179124 | Male | 8.11% | 1.47E-04 | Male | 8.51% |
| 1033 | 0.000171363 | Male | 7.65% | 1.47E-04 | Male | 8.49% |
| 1032 | 0.000174738 | Male | 7.85% | 1.47E-04 | Male | 8.49% |
| 1031 | 0.000170948 | Male | 7.63% | 1.47E-04 | Male | 8.49% |
| 1030 | 0.000172775 | Male | 7.74% | 1.47E-04 | Male | 8.49% |
| 1029 | 0.000171359 | Male | 7.65% | 1.47E-04 | Male | 8.46% |
| 1028 | 0.000180656 | Male | 8.20% | 1.46E-04 | Male | 8.45% |
| 1027 | 0.000176536 | Male | 7.96% | 1.46E-04 | Male | 8.44% |
| 1026 | 0.000167949 | Male | 7.45% | 1.46E-04 | Male | 8.44% |
| 1025 | 0.000172611 | Male | 7.73% | 1.46E-04 | Male | 8.42% |
| 1024 | 0.000170776 | Male | 7.62% | 1.46E-04 | Male | 8.41% |
| 1023 | 0.000173548 | Male | 7.78% | 1.46E-04 | Male | 8.40% |
| 1022 | 0.000168983 | Male | 7.51% | 1.46E-04 | Male | 8.40% |
| 1021 | 0.000170877 | Male | 7.62% | 1.46E-04 | Male | 8.40% |
| 1020 | 0.000168778 | Male | 7.50% | 1.45E-04 | Male | 8.38% |
| 1019 | 0.000171011 | Male | 7.63% | 1.45E-04 | Male | 8.35% |
| 1018 | 0.000173536 | Male | 7.78% | 1.44E-04 | Male | 8.32% |
| 1017 | 0.000166538 | Male | 7.37% | 1.44E-04 | Male | 8.31% |
| 1016 | 0.000168362 | Male | 7.47% | 1.44E-04 | Male | 8.31% |
| 1015 | 0.000170167 | Male | 7.58% | 1.44E-04 | Male | 8.31% |
| 1014 | 0.000171072 | Male | 7.64% | 1.44E-04 | Male | 8.31% |
| 1013 | 0.00017405 | Male | 7.81% | 1.44E-04 | Male | 8.30% |
| 1012 | 0.000170997 | Male | 7.63% | 1.44E-04 | Male | 8.28% |
| 1011 | 0.000174426 | Male | 7.83% | 1.43E-04 | Male | 8.28% |
| 1010 | 0.000175698 | Male | 7.91% | 1.43E-04 | Male | 8.27% |
| 1009 | 0.00017103 | Male | 7.63% | 1.43E-04 | Male | 8.27% |
| 1008 | 0.000168451 | Male | 7.48% | 1.43E-04 | Male | 8.27% |
| 1007 | 0.00016771 | Male | 7.44% | 1.43E-04 | Male | 8.26% |
| 1006 | 0.000169636 | Male | 7.55% | 1.43E-04 | Male | 8.24% |
| 1005 | 0.000172694 | Male | 7.73% | 1.42E-04 | Male | 8.22% |
| 1004 | 0.000170339 | Male | 7.59% | 1.42E-04 | Male | 8.21% |
| 1003 | 0.000171887 | Male | 7.68% | 1.42E-04 | Male | 8.21% |
| 1002 | 0.000169768 | Male | 7.56% | 1.42E-04 | Male | 8.20% |
| 1001 | 0.000165283 | Male | 7.29% | 1.42E-04 | Male | 8.18% |
| 1000 | 0.000166065 | Male | 7.34% | 1.42E-04 | Male | 8.16% |
| 999 | 0.000164964 | Male | 7.27% | 1.41E-04 | Male | 8.14% |
| 998 | 0.000167054 | Male | 7.40% | 1.41E-04 | Male | 8.13% |
| 997 | 0.00017096 | Male | 7.63% | 1.41E-04 | Male | 8.13% |
| 996 | 0.000168466 | Male | 7.48% | 1.40E-04 | Male | 8.10% |
| 995 | 0.000168248 | Male | 7.47% | 1.40E-04 | Male | 8.08% |
| 994 | 0.000164091 | Male | 7.22% | 1.40E-04 | Male | 8.06% |
| 993 | 0.000166346 | Male | 7.36% | 1.40E-04 | Male | 8.05% |
| 992 | 0.000170426 | Male | 7.60% | 1.39E-04 | Male | 7.99% |
| 991 | 0.00016534 | Male | 7.30% | 1.39E-04 | Male | 7.98% |
| 990 | 0.000162849 | Male | 7.15% | 1.38E-04 | Male | 7.98% |
| 989 | 0.000167523 | Male | 7.43% | 1.38E-04 | Male | 7.96% |
| 988 | 0.000167862 | Male | 7.45% | 1.38E-04 | Male | 7.96% |
| 987 | 0.000165197 | Male | 7.29% | 1.38E-04 | Male | 7.95% |
| 986 | 0.000165351 | Male | 7.30% | 1.38E-04 | Male | 7.93% |
| 985 | 0.000170715 | Male | 7.61% | 1.37E-04 | Male | 7.92% |
| 984 | 0.00016906 | Male | 7.52% | 1.37E-04 | Male | 7.88% |
| 983 | 0.000164824 | Male | 7.27% | 1.37E-04 | Male | 7.88% |
| 982 | 0.000167057 | Male | 7.40% | 1.37E-04 | Male | 7.87% |
| 981 | 0.000157084 | Male | 6.81% | 1.37E-04 | Male | 7.87% |
| 980 | 0.000161764 | Male | 7.08% | 1.37E-04 | Male | 7.87% |
| 979 | 0.000161569 | Male | 7.07% | 1.36E-04 | Male | 7.85% |
| 978 | 0.000157899 | Male | 6.86% | 1.36E-04 | Male | 7.81% |
| 977 | 0.000162125 | Male | 7.11% | 1.35E-04 | Male | 7.81% |
| 976 | 0.000161384 | Male | 7.06% | 1.35E-04 | Male | 7.79% |
| 975 | 0.000165411 | Male | 7.30% | 1.35E-04 | Male | 7.78% |
| 974 | 0.000160907 | Male | 7.03% | 1.35E-04 | Male | 7.77% |
| 973 | 0.00016504 | Male | 7.28% | 1.35E-04 | Male | 7.77% |
| 972 | 0.000159856 | Male | 6.97% | 1.35E-04 | Male | 7.76% |
| 971 | 0.000160535 | Male | 7.01% | 1.35E-04 | Male | 7.75% |
| 970 | 0.000163711 | Male | 7.20% | 1.34E-04 | Male | 7.75% |
| 969 | 0.000159626 | Male | 6.96% | 1.33E-04 | Male | 7.69% |
| 968 | 0.000158995 | Male | 6.92% | 1.33E-04 | Male | 7.69% |
| 967 | 0.000158555 | Male | 6.89% | 1.33E-04 | Male | 7.66% |
| 966 | 0.000160685 | Male | 7.02% | 1.33E-04 | Male | 7.65% |
| 965 | 0.000162217 | Male | 7.11% | 1.32E-04 | Male | 7.63% |
| 964 | 0.000163148 | Male | 7.17% | 1.32E-04 | Male | 7.57% |
| 963 | 0.000155246 | Male | 6.70% | 1.31E-04 | Male | 7.52% |
| 962 | 0.000158677 | Male | 6.90% | 1.30E-04 | Male | 7.50% |
| 961 | 0.000157943 | Male | 6.86% | 1.30E-04 | Male | 7.49% |
| 960 | 0.000158542 | Male | 6.89% | 1.30E-04 | Male | 7.49% |
| 959 | 0.000154667 | Male | 6.66% | 1.30E-04 | Male | 7.48% |
| 958 | 0.000158306 | Male | 6.88% | 1.30E-04 | Male | 7.48% |
| 957 | 0.000157687 | Male | 6.84% | 1.30E-04 | Male | 7.47% |
| 956 | 0.000151828 | Male | 6.50% | 1.29E-04 | Male | 7.45% |
| 955 | 0.000154911 | Male | 6.68% | 1.29E-04 | Male | 7.44% |
| 954 | 0.00015532 | Male | 6.70% | 1.29E-04 | Male | 7.43% |
| 953 | 0.000155555 | Male | 6.72% | 1.29E-04 | Male | 7.43% |
| 952 | 0.000151954 | Male | 6.50% | 1.29E-04 | Male | 7.42% |
| 951 | 0.000158896 | Male | 6.91% | 1.29E-04 | Male | 7.40% |
| 950 | 0.000154757 | Male | 6.67% | 1.29E-04 | Male | 7.40% |
| 949 | 0.000163429 | Male | 7.18% | 1.28E-04 | Male | 7.39% |
| 948 | 0.00015547 | Male | 6.71% | 1.28E-04 | Male | 7.35% |
| 947 | 0.000151853 | Male | 6.50% | 1.28E-04 | Male | 7.34% |
| 946 | 0.00015243 | Male | 6.53% | 1.27E-04 | Male | 7.32% |
| 945 | 0.000152383 | Male | 6.53% | 1.27E-04 | Male | 7.32% |
| 944 | 0.000159184 | Male | 6.93% | 1.27E-04 | Male | 7.32% |
| 943 | 0.000158708 | Male | 6.90% | 1.27E-04 | Male | 7.32% |
| 942 | 0.000150441 | Male | 6.41% | 1.27E-04 | Male | 7.32% |
| 941 | 0.000156078 | Male | 6.75% | 1.27E-04 | Male | 7.31% |
| 940 | 0.000158485 | Male | 6.89% | 1.27E-04 | Male | 7.30% |
| 939 | 0.000153188 | Male | 6.58% | 1.27E-04 | Male | 7.30% |
| 938 | 0.0001545 | Male | 6.65% | 1.26E-04 | Male | 7.26% |
| 937 | 0.000152475 | Male | 6.53% | 1.26E-04 | Male | 7.25% |
| 936 | 0.000154986 | Male | 6.68% | 1.26E-04 | Male | 7.25% |
| 935 | 0.00014905 | Male | 6.33% | 1.26E-04 | Male | 7.22% |
| 934 | 0.000153168 | Male | 6.57% | 1.26E-04 | Male | 7.22% |
| 933 | 0.000149433 | Male | 6.35% | 1.25E-04 | Male | 7.18% |
| 932 | 0.000154955 | Male | 6.68% | 1.25E-04 | Male | 7.17% |
| 931 | 0.000151363 | Male | 6.47% | 1.25E-04 | Male | 7.17% |
| 930 | 0.000149695 | Male | 6.37% | 1.24E-04 | Male | 7.15% |
| 929 | 0.000152182 | Male | 6.52% | 1.24E-04 | Male | 7.11% |
| 928 | 0.00014746 | Male | 6.24% | 1.24E-04 | Male | 7.10% |
| 927 | 0.000153099 | Male | 6.57% | 1.23E-04 | Male | 7.10% |
| 926 | 0.000145473 | Male | 6.12% | 1.23E-04 | Male | 7.09% |
| 925 | 0.000149525 | Male | 6.36% | 1.23E-04 | Male | 7.09% |
| 924 | 0.000145307 | Male | 6.11% | 1.23E-04 | Male | 7.08% |
| 923 | 0.000144423 | Male | 6.06% | 1.23E-04 | Male | 7.06% |
| 922 | 0.000147235 | Male | 6.22% | 1.23E-04 | Male | 7.05% |
| 921 | 0.000148899 | Male | 6.32% | 1.23E-04 | Male | 7.04% |
| 920 | 0.000156318 | Male | 6.76% | 1.22E-04 | Male | 7.01% |
| 919 | 0.000149539 | Male | 6.36% | 1.22E-04 | Male | 7.01% |
| 918 | 0.000151787 | Male | 6.49% | 1.21E-04 | Male | 6.97% |
| 917 | 0.000141443 | Male | 5.88% | 1.21E-04 | Male | 6.96% |
| 916 | 0.000148554 | Male | 6.30% | 1.21E-04 | Male | 6.95% |
| 915 | 0.000147615 | Male | 6.25% | 1.21E-04 | Male | 6.94% |
| 914 | 0.000151731 | Male | 6.49% | 1.21E-04 | Male | 6.93% |
| 913 | 0.000149566 | Male | 6.36% | 1.20E-04 | Male | 6.89% |
| 912 | 0.000143424 | Male | 6.00% | 1.20E-04 | Male | 6.89% |
| 911 | 0.000146347 | Male | 6.17% | 1.20E-04 | Male | 6.88% |
| 910 | 0.000137693 | Male | 5.66% | 1.20E-04 | Male | 6.87% |
| 909 | 0.000145792 | Male | 6.14% | 1.19E-04 | Male | 6.84% |
| 908 | 0.000144907 | Male | 6.09% | 1.19E-04 | Male | 6.84% |
| 907 | 0.000147531 | Male | 6.24% | 1.19E-04 | Male | 6.83% |
| 906 | 0.000144201 | Male | 6.04% | 1.19E-04 | Male | 6.81% |
| 905 | 0.0001493 | Male | 6.35% | 1.18E-04 | Male | 6.79% |
| 904 | 0.000137758 | Male | 5.66% | 1.17E-04 | Male | 6.72% |
| 903 | 0.000144585 | Male | 6.07% | 1.17E-04 | Male | 6.72% |
| 902 | 0.000141277 | Male | 5.87% | 1.17E-04 | Male | 6.71% |
| 901 | 0.00014266 | Male | 5.95% | 1.17E-04 | Male | 6.70% |
| 900 | 0.000140485 | Male | 5.82% | 1.17E-04 | Male | 6.69% |
| 899 | 0.000139599 | Male | 5.77% | 1.17E-04 | Male | 6.69% |
| 898 | 0.000142074 | Male | 5.92% | 1.16E-04 | Male | 6.67% |
| 897 | 0.000139543 | Male | 5.77% | 1.16E-04 | Male | 6.64% |
| 896 | 0.000137157 | Male | 5.63% | 1.15E-04 | Male | 6.61% |
| 895 | 0.000134568 | Male | 5.47% | 1.15E-04 | Male | 6.59% |
| 894 | 0.000138072 | Male | 5.68% | 1.15E-04 | Male | 6.58% |
| 893 | 0.000142216 | Male | 5.93% | 1.14E-04 | Male | 6.54% |
| 892 | 0.000142756 | Male | 5.96% | 1.14E-04 | Male | 6.54% |
| 891 | 0.000144845 | Male | 6.08% | 1.14E-04 | Male | 6.52% |
| 890 | 0.000138167 | Male | 5.69% | 1.13E-04 | Male | 6.49% |
| 889 | 0.000141308 | Male | 5.87% | 1.13E-04 | Male | 6.48% |
| 888 | 0.000136113 | Male | 5.56% | 1.13E-04 | Male | 6.46% |
| 887 | 0.000135509 | Male | 5.53% | 1.12E-04 | Male | 6.45% |
| 886 | 0.000135841 | Male | 5.55% | 1.12E-04 | Male | 6.43% |
| 885 | 0.000141223 | Male | 5.87% | 1.12E-04 | Male | 6.39% |
| 884 | 0.000134849 | Male | 5.49% | 1.11E-04 | Male | 6.38% |
| 883 | 0.000137134 | Male | 5.62% | 1.11E-04 | Male | 6.38% |
| 882 | 0.000136976 | Male | 5.62% | 1.11E-04 | Male | 6.35% |
| 881 | 0.000133985 | Male | 5.44% | 1.10E-04 | Male | 6.31% |
| 880 | 0.000133824 | Male | 5.43% | 1.10E-04 | Male | 6.31% |
| 879 | 0.000134272 | Male | 5.46% | 1.09E-04 | Male | 6.25% |
| 878 | 0.000132142 | Male | 5.33% | 1.09E-04 | Male | 6.25% |
| 877 | 0.000134731 | Male | 5.48% | 1.09E-04 | Male | 6.24% |
| 876 | 0.000132916 | Male | 5.37% | 1.08E-04 | Male | 6.20% |
| 875 | 0.000132622 | Male | 5.36% | 1.08E-04 | Male | 6.19% |
| 874 | 0.000126261 | Male | 4.98% | 1.08E-04 | Male | 6.17% |
| 873 | 0.000130965 | Male | 5.26% | 1.08E-04 | Male | 6.16% |
| 872 | 0.000131408 | Male | 5.29% | 1.07E-04 | Male | 6.16% |
| 871 | 0.000134836 | Male | 5.49% | 1.07E-04 | Male | 6.14% |
| 870 | 0.000134642 | Male | 5.48% | 1.07E-04 | Male | 6.11% |
| 869 | 0.000132033 | Male | 5.32% | 1.06E-04 | Male | 6.07% |
| 868 | 0.00012723 | Male | 5.04% | 1.06E-04 | Male | 6.04% |
| 867 | 0.000132355 | Male | 5.34% | 1.06E-04 | Male | 6.04% |
| 866 | 0.000129302 | Male | 5.16% | 1.05E-04 | Male | 6.01% |
| 865 | 0.000131544 | Male | 5.29% | 1.05E-04 | Male | 5.99% |
| 864 | 0.000127757 | Male | 5.07% | 1.05E-04 | Male | 5.98% |
| 863 | 0.000124371 | Male | 4.87% | 1.04E-04 | Male | 5.97% |
| 862 | 0.00012598 | Male | 4.96% | 1.04E-04 | Male | 5.94% |
| 861 | 0.000124459 | Male | 4.87% | 1.03E-04 | Male | 5.90% |
| 860 | 0.000125423 | Male | 4.93% | 1.03E-04 | Male | 5.89% |
| 859 | 0.000129174 | Male | 5.15% | 1.03E-04 | Male | 5.87% |
| 858 | 0.000126775 | Male | 5.01% | 1.02E-04 | Male | 5.85% |
| 857 | 0.000129982 | Male | 5.20% | 1.02E-04 | Male | 5.84% |
| 856 | 0.000123686 | Male | 4.83% | 1.02E-04 | Male | 5.82% |
| 855 | 0.000127313 | Male | 5.04% | 1.01E-04 | Male | 5.80% |
| 854 | 0.00012773 | Male | 5.07% | 1.01E-04 | Male | 5.78% |
| 853 | 0.000124638 | Male | 4.88% | 1.01E-04 | Male | 5.78% |
| 852 | 0.000126146 | Male | 4.97% | 1.01E-04 | Male | 5.77% |
| 851 | 0.000126766 | Male | 5.01% | 1.01E-04 | Male | 5.75% |
| 850 | 0.000127941 | Male | 5.08% | 1.00E-04 | Male | 5.74% |
| 849 | 0.000126005 | Male | 4.97% | 9.93E-05 | Male | 5.67% |
| 848 | 0.000123136 | Male | 4.80% | 9.85E-05 | Male | 5.63% |
| 847 | 0.000126507 | Male | 5.00% | 9.80E-05 | Male | 5.60% |
| 846 | 0.000130062 | Male | 5.21% | 9.76E-05 | Male | 5.57% |
| 845 | 0.000122836 | Male | 4.78% | 9.74E-05 | Male | 5.56% |
| 844 | 0.00012149 | Male | 4.70% | 9.73E-05 | Male | 5.56% |
| 843 | 0.000126531 | Male | 5.00% | 9.56E-05 | Male | 5.46% |
| 842 | 0.000117492 | Male | 4.46% | 9.50E-05 | Male | 5.42% |
| 841 | 0.000116409 | Male | 4.40% | 9.24E-05 | Male | 5.27% |
| 840 | 0.000114862 | Male | 4.31% | 9.16E-05 | Male | 5.22% |
| 839 | 0.000116171 | Male | 4.38% | 9.01E-05 | Male | 5.13% |
| 838 | 0.00011329 | Male | 4.21% | 8.96E-05 | Male | 5.10% |
| 837 | 0.000105747 | Male | 3.77% | 8.82E-05 | Male | 5.02% |
| 836 | 0.000119639 | Male | 4.59% | 8.82E-05 | Male | 5.02% |
| 835 | 0.000108552 | Male | 3.93% | 8.81E-05 | Male | 5.01% |
| 834 | 0.000111362 | Male | 4.10% | 8.80E-05 | Male | 5.01% |
| 833 | 0.000114401 | Male | 4.28% | 8.80E-05 | Male | 5.01% |
| 832 | 0.000113158 | Male | 4.20% | 8.68E-05 | Male | 4.94% |
| 831 | 0.000111714 | Male | 4.12% | 8.65E-05 | Male | 4.92% |
| 830 | 0.000112026 | Male | 4.14% | 8.57E-05 | Male | 4.87% |
| 829 | 0.000107805 | Male | 3.89% | 8.54E-05 | Male | 4.85% |
| 828 | 0.000112238 | Male | 4.15% | 8.49E-05 | Male | 4.83% |
| 827 | 0.00010761 | Male | 3.88% | 8.29E-05 | Male | 4.71% |
| 826 | 0.000103061 | Male | 3.61% | 8.27E-05 | Male | 4.69% |
| 825 | 0.000108743 | Male | 3.94% | 8.19E-05 | Male | 4.65% |
| 824 | 0.000113605 | Male | 4.23% | 8.18E-05 | Male | 4.64% |
| 823 | 0.000107255 | Male | 3.85% | 7.96E-05 | Male | 4.51% |
| 822 | 0.000107022 | Male | 3.84% | 7.82E-05 | Male | 4.43% |
| 821 | 0.000102722 | Male | 3.59% | 7.81E-05 | Male | 4.43% |
| 820 | 9.92E-05 | Male | 3.38% | 7.74E-05 | Male | 4.38% |
| 819 | 9.80E-05 | Male | 3.31% | 7.65E-05 | Male | 4.33% |
| 818 | 1.01E-04 | Male | 3.51% | 7.63E-05 | Male | 4.32% |
| 817 | 1.06E-04 | Male | 3.78% | 7.52E-05 | Male | 4.25% |
| 816 | 1.04E-04 | Male | 3.65% | 7.29E-05 | Male | 4.12% |
| 815 | 9.84E-05 | Male | 3.33% | 7.25E-05 | Male | 4.09% |
| 814 | 9.73E-05 | Male | 3.27% | 7.20E-05 | Male | 4.06% |
| 813 | 9.54E-05 | Male | 3.15% | 7.08E-05 | Male | 3.99% |
| 812 | 9.65E-05 | Male | 3.22% | 6.94E-05 | Male | 3.91% |
| 811 | 8.83E-05 | Male | 2.73% | 6.71E-05 | Male | 3.77% |
| 810 | 9.37E-05 | Male | 3.05% | 6.67E-05 | Male | 3.75% |
| 809 | 9.03E-05 | Male | 2.85% | 6.40E-05 | Male | 3.60% |
| 808 | 8.87E-05 | Male | 2.75% | 5.98E-05 | Male | 3.35% |
| 807 | 8.10E-05 | Male | 2.30% | 5.75E-05 | Male | 3.21% |
| 806 | 8.49E-05 | Male | 2.53% | 5.65E-05 | Male | 3.15% |
| 805 | 7.54E-05 | Male | 1.97% | 5.24E-05 | Male | 2.91% |
| 804 | 7.82E-05 | Male | 2.13% | 5.22E-05 | Male | 2.90% |
| 803 | 6.93E-05 | Male | 1.61% | 4.37E-05 | Male | 2.39% |
| 802 | 6.48E-05 | Male | 1.34% | 4.01E-05 | Male | 2.18% |
| 801 | 6.73E-05 | Male | 1.49% | 3.82E-05 | Male | 2.08% |
| 800 | 6.77E-05 | Male | 1.51% | 3.21E-05 | Male | 1.72% |
| 799 | 6.43E-05 | Male | 1.31% | 3.18E-05 | Male | 1.69% |
| 798 | 5.62E-05 | Female |  | 2.70E-05 | Male | 1.41% |
| 797 | 7.61E-05 | Male | 2.01% | 2.45E-05 | Male | 1.26% |
| 796 | 6.34E-05 | Male | 1.26% | 2.41E-05 | Male | 1.24% |
| 795 | 4.92E-05 | Female |  | 2.38E-05 | Male | 1.22% |
| 1 | 2.50E-05 | Female |  | 1.02E-06 | Female |  |
| 2 | 1.93E-05 | Female |  | 1.05E-06 | Female |  |
| 3 | 2.86E-05 | Female |  | 1.12E-06 | Female |  |
| 4 | 2.32E-05 | Female |  | 1.12E-06 | Female |  |
| 5 | 2.88E-05 | Female |  | 1.13E-06 | Female |  |
| 6 | 2.62E-05 | Female |  | 1.15E-06 | Female |  |
| 7 | 2.63E-05 | Female |  | 1.15E-06 | Female |  |
| 8 | 2.68E-05 | Female |  | 1.16E-06 | Female |  |
| 9 | 2.16E-05 | Female |  | 1.17E-06 | Female |  |
| 10 | 2.65E-05 | Female |  | 1.20E-06 | Female |  |
| 11 | 2.41E-05 | Female |  | 1.22E-06 | Female |  |
| 12 | 2.52E-05 | Female |  | 1.25E-06 | Female |  |
| 13 | 2.42E-05 | Female |  | 1.27E-06 | Female |  |
| 14 | 2.36E-05 | Female |  | 1.30E-06 | Female |  |
| 15 | 2.33E-05 | Female |  | 1.31E-06 | Female |  |
| 16 | 2.72E-05 | Female |  | 1.31E-06 | Female |  |
| 17 | 2.36E-05 | Female |  | 1.34E-06 | Female |  |
| 18 | 2.67E-05 | Female |  | 1.34E-06 | Female |  |
| 19 | 2.48E-05 | Female |  | 1.36E-06 | Female |  |
| 20 | 2.57E-05 | Female |  | 1.39E-06 | Female |  |
| 21 | 2.77E-05 | Female |  | 1.40E-06 | Female |  |
| 22 | 1.99E-05 | Female |  | 1.40E-06 | Female |  |
| 23 | 2.58E-05 | Female |  | 1.43E-06 | Female |  |
| 24 | 2.61E-05 | Female |  | 1.43E-06 | Female |  |
| 25 | 2.27E-05 | Female |  | 1.44E-06 | Female |  |
| 26 | 2.32E-05 | Female |  | 1.45E-06 | Female |  |
| 27 | 3.05E-05 | Female |  | 1.46E-06 | Female |  |
| 28 | 1.94E-05 | Female |  | 1.48E-06 | Female |  |
| 29 | 2.40E-05 | Female |  | 1.51E-06 | Female |  |
| 30 | 2.84E-05 | Female |  | 1.51E-06 | Female |  |
| 31 | 2.47E-05 | Female |  | 1.55E-06 | Female |  |
| 32 | 2.75E-05 | Female |  | 1.57E-06 | Female |  |
| 33 | 2.81E-05 | Female |  | 1.58E-06 | Female |  |
| 34 | 2.74E-05 | Female |  | 1.62E-06 | Female |  |
| 35 | 2.21E-05 | Female |  | 1.62E-06 | Female |  |
| 36 | 2.45E-05 | Female |  | 1.65E-06 | Female |  |
| 37 | 2.10E-05 | Female |  | 1.65E-06 | Female |  |
| 38 | 2.17E-05 | Female |  | 1.68E-06 | Female |  |
| 39 | 2.67E-05 | Female |  | 1.71E-06 | Female |  |
| 40 | 2.93E-05 | Female |  | 1.71E-06 | Female |  |
| 41 | 2.59E-05 | Female |  | 1.72E-06 | Female |  |
| 42 | 2.64E-05 | Female |  | 1.72E-06 | Female |  |
| 43 | 2.77E-05 | Female |  | 1.72E-06 | Female |  |
| 44 | 2.89E-05 | Female |  | 1.74E-06 | Female |  |
| 45 | 2.93E-05 | Female |  | 1.74E-06 | Female |  |
| 46 | 2.79E-05 | Female |  | 1.75E-06 | Female |  |
| 47 | 2.17E-05 | Female |  | 1.75E-06 | Female |  |
| 48 | 2.47E-05 | Female |  | 1.76E-06 | Female |  |
| 49 | 2.43E-05 | Female |  | 1.76E-06 | Female |  |
| 50 | 2.47E-05 | Female |  | 1.78E-06 | Female |  |
| 51 | 2.79E-05 | Female |  | 1.80E-06 | Female |  |
| 52 | 2.48E-05 | Female |  | 1.81E-06 | Female |  |
| 53 | 2.92E-05 | Female |  | 1.82E-06 | Female |  |
| 54 | 2.37E-05 | Female |  | 1.83E-06 | Female |  |
| 55 | 1.87E-05 | Female |  | 1.87E-06 | Female |  |
| 56 | 2.45E-05 | Female |  | 1.90E-06 | Female |  |
| 57 | 2.90E-05 | Female |  | 1.90E-06 | Female |  |
| 58 | 2.51E-05 | Female |  | 1.91E-06 | Female |  |
| 59 | 3.03E-05 | Female |  | 1.92E-06 | Female |  |
| 60 | 2.78E-05 | Female |  | 1.92E-06 | Female |  |
| 61 | 2.86E-05 | Female |  | 1.93E-06 | Female |  |
| 62 | 2.48E-05 | Female |  | 1.93E-06 | Female |  |
| 63 | 2.54E-05 | Female |  | 1.94E-06 | Female |  |
| 64 | 2.37E-05 | Female |  | 1.95E-06 | Female |  |
| 65 | 2.83E-05 | Female |  | 1.95E-06 | Female |  |
| 66 | 2.47E-05 | Female |  | 1.98E-06 | Female |  |
| 67 | 2.27E-05 | Female |  | 1.98E-06 | Female |  |
| 68 | 2.25E-05 | Female |  | 2.00E-06 | Female |  |
| 69 | 2.46E-05 | Female |  | 2.02E-06 | Female |  |
| 70 | 2.23E-05 | Female |  | 2.02E-06 | Female |  |
| 71 | 2.21E-05 | Female |  | 2.02E-06 | Female |  |
| 72 | 2.55E-05 | Female |  | 2.03E-06 | Female |  |
| 73 | 2.56E-05 | Female |  | 2.04E-06 | Female |  |
| 74 | 2.40E-05 | Female |  | 2.04E-06 | Female |  |
| 75 | 2.34E-05 | Female |  | 2.04E-06 | Female |  |
| 76 | 2.79E-05 | Female |  | 2.04E-06 | Female |  |
| 77 | 2.55E-05 | Female |  | 2.05E-06 | Female |  |
| 78 | 1.85E-05 | Female |  | 2.07E-06 | Female |  |
| 79 | 2.01E-05 | Female |  | 2.10E-06 | Female |  |
| 80 | 2.55E-05 | Female |  | 2.10E-06 | Female |  |
| 81 | 3.57E-05 | Female |  | 8.72E-06 | Female |  |
| 82 | 2.28E-05 | Female |  | 2.16E-06 | Female |  |
| 83 | 2.46E-05 | Female |  | 2.16E-06 | Female |  |
| 84 | 2.40E-05 | Female |  | 2.16E-06 | Female |  |
| 85 | 2.95E-05 | Female |  | 1.07E-05 | Female |  |
| 86 | 2.61E-05 | Female |  | 2.23E-06 | Female |  |
| 87 | 2.12E-05 | Female |  | 2.23E-06 | Female |  |
| 88 | 2.56E-05 | Female |  | 2.25E-06 | Female |  |
| 89 | 2.46E-05 | Female |  | 2.26E-06 | Female |  |
| 90 | 2.49E-05 | Female |  | 2.27E-06 | Female |  |
| 91 | 2.38E-05 | Female |  | 2.27E-06 | Female |  |
| 92 | 2.66E-05 | Female |  | 2.29E-06 | Female |  |
| 93 | 2.58E-05 | Female |  | 2.29E-06 | Female |  |
| 94 | 3.08E-05 | Female |  | 2.30E-06 | Female |  |
| 95 | 2.48E-05 | Female |  | 2.30E-06 | Female |  |
| 96 | 2.98E-05 | Female |  | 2.34E-06 | Female |  |
| 97 | 2.59E-05 | Female |  | 2.35E-06 | Female |  |
| 98 | 2.27E-05 | Female |  | 2.35E-06 | Female |  |
| 99 | 4.20E-05 | Female |  | 1.77E-05 | Female |  |
| 100 | 2.77E-05 | Female |  | 2.38E-06 | Female |  |
| 101 | 2.46E-05 | Female |  | 2.38E-06 | Female |  |
| 102 | 2.36E-05 | Female |  | 2.39E-06 | Female |  |
| 103 | 2.83E-05 | Female |  | 2.39E-06 | Female |  |
| 104 | 2.68E-05 | Female |  | 2.39E-06 | Female |  |
| 105 | 2.61E-05 | Female |  | 2.40E-06 | Female |  |
| 106 | 2.38E-05 | Female |  | 2.42E-06 | Female |  |
| 107 | 2.43E-05 | Female |  | 2.43E-06 | Female |  |
| 108 | 2.61E-05 | Female |  | 2.44E-06 | Female |  |
| 109 | 2.40E-05 | Female |  | 2.45E-06 | Female |  |
| 110 | 2.33E-05 | Female |  | 2.45E-06 | Female |  |
| 111 | 2.46E-05 | Female |  | 2.46E-06 | Female |  |
| 112 | 2.59E-05 | Female |  | 2.50E-06 | Female |  |
| 113 | 2.60E-05 | Female |  | 2.50E-06 | Female |  |
| 114 | 2.37E-05 | Female |  | 2.52E-06 | Female |  |
| 115 | 2.90E-05 | Female |  | 2.52E-06 | Female |  |
| 116 | 2.54E-05 | Female |  | 2.53E-06 | Female |  |
| 117 | 2.62E-05 | Female |  | 2.55E-06 | Female |  |
| 118 | 2.60E-05 | Female |  | 2.57E-06 | Female |  |
| 119 | 2.63E-05 | Female |  | 2.58E-06 | Female |  |
| 120 | 2.68E-05 | Female |  | 2.58E-06 | Female |  |
| 121 | 2.36E-05 | Female |  | 2.60E-06 | Female |  |
| 122 | 3.38E-05 | Female |  | 2.60E-06 | Female |  |
| 123 | 2.54E-05 | Female |  | 2.60E-06 | Female |  |
| 124 | 2.45E-05 | Female |  | 2.61E-06 | Female |  |
| 125 | 3.02E-05 | Female |  | 2.62E-06 | Female |  |
| 126 | 2.64E-05 | Female |  | 2.62E-06 | Female |  |
| 127 | 2.60E-05 | Female |  | 2.62E-06 | Female |  |
| 128 | 2.70E-05 | Female |  | 2.63E-06 | Female |  |
| 129 | 3.99E-05 | Female |  | 1.26E-05 | Female |  |
| 130 | 2.87E-05 | Female |  | 2.65E-06 | Female |  |
| 131 | 2.29E-05 | Female |  | 2.66E-06 | Female |  |
| 132 | 2.93E-05 | Female |  | 2.67E-06 | Female |  |
| 133 | 2.65E-05 | Female |  | 2.68E-06 | Female |  |
| 134 | 2.49E-05 | Female |  | 2.68E-06 | Female |  |
| 135 | 2.35E-05 | Female |  | 2.71E-06 | Female |  |
| 136 | 2.31E-05 | Female |  | 2.72E-06 | Female |  |
| 137 | 2.77E-05 | Female |  | 2.72E-06 | Female |  |
| 138 | 2.84E-05 | Female |  | 2.74E-06 | Female |  |
| 139 | 2.89E-05 | Female |  | 2.75E-06 | Female |  |
| 140 | 2.54E-05 | Female |  | 2.76E-06 | Female |  |
| 141 | 2.62E-05 | Female |  | 2.77E-06 | Female |  |
| 142 | 2.69E-05 | Female |  | 2.77E-06 | Female |  |
| 143 | 2.82E-05 | Female |  | 2.79E-06 | Female |  |
| 144 | 2.40E-05 | Female |  | 2.79E-06 | Female |  |
| 145 | 2.17E-05 | Female |  | 2.79E-06 | Female |  |
| 146 | 2.79E-05 | Female |  | 2.80E-06 | Female |  |
| 147 | 3.26E-05 | Female |  | 2.80E-06 | Female |  |
| 148 | 3.19E-05 | Female |  | 1.04E-06 | Female |  |
| 149 | 2.90E-05 | Female |  | 2.84E-06 | Female |  |
| 150 | 2.72E-05 | Female |  | 2.84E-06 | Female |  |
| 151 | 3.12E-05 | Female |  | 2.85E-06 | Female |  |
| 152 | 2.29E-05 | Female |  | 2.86E-06 | Female |  |
| 153 | 2.92E-05 | Female |  | 2.86E-06 | Female |  |
| 154 | 2.84E-05 | Female |  | 2.86E-06 | Female |  |
| 155 | 2.18E-05 | Female |  | 2.88E-06 | Female |  |
| 156 | 2.96E-05 | Female |  | 2.89E-06 | Female |  |
| 157 | 2.76E-05 | Female |  | 2.95E-06 | Female |  |
| 158 | 2.73E-05 | Female |  | 2.95E-06 | Female |  |
| 159 | 2.73E-05 | Female |  | 2.96E-06 | Female |  |
| 160 | 3.03E-05 | Female |  | 2.99E-06 | Female |  |
| 161 | 2.50E-05 | Female |  | 3.00E-06 | Female |  |
| 162 | 3.03E-05 | Female |  | 3.02E-06 | Female |  |
| 163 | 2.42E-05 | Female |  | 3.02E-06 | Female |  |
| 164 | 3.04E-05 | Female |  | 3.03E-06 | Female |  |
| 165 | 2.58E-05 | Female |  | 3.04E-06 | Female |  |
| 166 | 4.00E-05 | Female |  | 1.47E-05 | Female |  |
| 167 | 2.48E-05 | Female |  | 3.06E-06 | Female |  |
| 168 | 2.60E-05 | Female |  | 3.07E-06 | Female |  |
| 169 | 2.65E-05 | Female |  | 3.07E-06 | Female |  |
| 170 | 3.35E-05 | Female |  | 3.09E-06 | Female |  |
| 171 | 2.68E-05 | Female |  | 3.10E-06 | Female |  |
| 172 | 3.04E-05 | Female |  | 3.11E-06 | Female |  |
| 173 | 3.10E-05 | Female |  | 3.11E-06 | Female |  |
| 174 | 3.02E-05 | Female |  | 3.12E-06 | Female |  |
| 175 | 2.71E-05 | Female |  | 3.13E-06 | Female |  |
| 176 | 2.31E-05 | Female |  | 3.13E-06 | Female |  |
| 177 | 2.83E-05 | Female |  | 3.19E-06 | Female |  |
| 178 | 2.38E-05 | Female |  | 3.19E-06 | Female |  |
| 179 | 2.69E-05 | Female |  | 3.20E-06 | Female |  |
| 180 | 2.64E-05 | Female |  | 3.20E-06 | Female |  |
| 181 | 2.74E-05 | Female |  | 3.20E-06 | Female |  |
| 182 | 2.54E-05 | Female |  | 3.21E-06 | Female |  |
| 183 | 2.44E-05 | Female |  | 3.21E-06 | Female |  |
| 184 | 2.73E-05 | Female |  | 3.23E-06 | Female |  |
| 185 | 2.21E-05 | Female |  | 3.25E-06 | Female |  |
| 186 | 2.99E-05 | Female |  | 3.26E-06 | Female |  |
| 187 | 2.75E-05 | Female |  | 3.27E-06 | Female |  |
| 188 | 2.68E-05 | Female |  | 3.29E-06 | Female |  |
| 189 | 3.26E-05 | Female |  | 3.30E-06 | Female |  |
| 190 | 2.55E-05 | Female |  | 3.31E-06 | Female |  |
| 191 | 2.82E-05 | Female |  | 3.32E-06 | Female |  |
| 192 | 2.26E-05 | Female |  | 3.33E-06 | Female |  |
| 193 | 2.36E-05 | Female |  | 3.34E-06 | Female |  |
| 194 | 2.92E-05 | Female |  | 3.34E-06 | Female |  |
| 195 | 3.11E-05 | Female |  | 3.34E-06 | Female |  |
| 196 | 2.37E-05 | Female |  | 3.36E-06 | Female |  |
| 197 | 2.61E-05 | Female |  | 3.37E-06 | Female |  |
| 198 | 2.79E-05 | Female |  | 3.38E-06 | Female |  |
| 199 | 2.33E-05 | Female |  | 3.40E-06 | Female |  |
| 200 | 2.19E-05 | Female |  | 3.40E-06 | Female |  |
| 201 | 2.43E-05 | Female |  | 3.40E-06 | Female |  |
| 202 | 2.20E-05 | Female |  | 3.41E-06 | Female |  |
| 203 | 2.50E-05 | Female |  | 3.42E-06 | Female |  |
| 204 | 2.87E-05 | Female |  | 3.42E-06 | Female |  |
| 205 | 2.77E-05 | Female |  | 3.42E-06 | Female |  |
| 206 | 3.01E-05 | Female |  | 3.42E-06 | Female |  |
| 207 | 3.17E-05 | Female |  | 3.43E-06 | Female |  |
| 208 | 2.31E-05 | Female |  | 3.44E-06 | Female |  |
| 209 | 2.50E-05 | Female |  | 3.45E-06 | Female |  |
| 210 | 2.48E-05 | Female |  | 3.49E-06 | Female |  |
| 211 | 2.56E-05 | Female |  | 3.49E-06 | Female |  |
| 212 | 2.75E-05 | Female |  | 3.49E-06 | Female |  |
| 213 | 2.92E-05 | Female |  | 3.50E-06 | Female |  |
| 214 | 2.46E-05 | Female |  | 3.50E-06 | Female |  |
| 215 | 3.17E-05 | Female |  | 3.51E-06 | Female |  |
| 216 | 2.75E-05 | Female |  | 3.51E-06 | Female |  |
| 217 | 2.64E-05 | Female |  | 3.51E-06 | Female |  |
| 218 | 2.44E-05 | Female |  | 3.52E-06 | Female |  |
| 219 | 2.32E-05 | Female |  | 3.52E-06 | Female |  |
| 220 | 2.78E-05 | Female |  | 3.55E-06 | Female |  |
| 221 | 2.70E-05 | Female |  | 3.56E-06 | Female |  |
| 222 | 2.76E-05 | Female |  | 3.56E-06 | Female |  |
| 223 | 2.57E-05 | Female |  | 3.57E-06 | Female |  |
| 224 | 2.66E-05 | Female |  | 3.57E-06 | Female |  |
| 225 | 3.01E-05 | Female |  | 3.58E-06 | Female |  |
| 226 | 2.60E-05 | Female |  | 3.58E-06 | Female |  |
| 227 | 2.78E-05 | Female |  | 3.60E-06 | Female |  |
| 228 | 3.52E-05 | Female |  | 3.61E-06 | Female |  |
| 229 | 2.37E-05 | Female |  | 3.62E-06 | Female |  |
| 230 | 2.72E-05 | Female |  | 3.63E-06 | Female |  |
| 231 | 2.73E-05 | Female |  | 3.63E-06 | Female |  |
| 232 | 2.08E-05 | Female |  | 3.63E-06 | Female |  |
| 233 | 2.70E-05 | Female |  | 3.64E-06 | Female |  |
| 234 | 3.03E-05 | Female |  | 3.65E-06 | Female |  |
| 235 | 2.95E-05 | Female |  | 3.65E-06 | Female |  |
| 236 | 2.36E-05 | Female |  | 3.66E-06 | Female |  |
| 237 | 3.84E-05 | Female |  | 1.33E-05 | Female |  |
| 238 | 2.72E-05 | Female |  | 3.67E-06 | Female |  |
| 239 | 2.49E-05 | Female |  | 3.69E-06 | Female |  |
| 240 | 2.73E-05 | Female |  | 3.70E-06 | Female |  |
| 241 | 2.81E-05 | Female |  | 3.71E-06 | Female |  |
| 242 | 2.74E-05 | Female |  | 3.72E-06 | Female |  |
| 243 | 2.94E-05 | Female |  | 3.72E-06 | Female |  |
| 244 | 2.69E-05 | Female |  | 3.73E-06 | Female |  |
| 245 | 3.04E-05 | Female |  | 3.75E-06 | Female |  |
| 246 | 2.36E-05 | Female |  | 3.76E-06 | Female |  |
| 247 | 2.82E-05 | Female |  | 3.76E-06 | Female |  |
| 248 | 2.69E-05 | Female |  | 3.76E-06 | Female |  |
| 249 | 2.42E-05 | Female |  | 3.77E-06 | Female |  |
| 250 | 2.64E-05 | Female |  | 3.77E-06 | Female |  |
| 251 | 2.45E-05 | Female |  | 3.80E-06 | Female |  |
| 252 | 2.59E-05 | Female |  | 3.80E-06 | Female |  |
| 253 | 3.23E-05 | Female |  | 3.81E-06 | Female |  |
| 254 | 2.48E-05 | Female |  | 3.81E-06 | Female |  |
| 255 | 3.28E-05 | Female |  | 3.82E-06 | Female |  |
| 256 | 2.83E-05 | Female |  | 3.82E-06 | Female |  |
| 257 | 2.98E-05 | Female |  | 3.83E-06 | Female |  |
| 258 | 3.03E-05 | Female |  | 3.83E-06 | Female |  |
| 259 | 2.74E-05 | Female |  | 3.83E-06 | Female |  |
| 260 | 3.21E-05 | Female |  | 3.83E-06 | Female |  |
| 261 | 2.92E-05 | Female |  | 3.83E-06 | Female |  |
| 262 | 2.81E-05 | Female |  | 3.84E-06 | Female |  |
| 263 | 2.60E-05 | Female |  | 3.84E-06 | Female |  |
| 264 | 3.48E-05 | Female |  | 3.85E-06 | Female |  |
| 265 | 3.33E-05 | Female |  | 3.85E-06 | Female |  |
| 266 | 3.27E-05 | Female |  | 3.86E-06 | Female |  |
| 267 | 2.53E-05 | Female |  | 3.86E-06 | Female |  |
| 268 | 2.66E-05 | Female |  | 3.86E-06 | Female |  |
| 269 | 2.79E-05 | Female |  | 3.88E-06 | Female |  |
| 270 | 2.58E-05 | Female |  | 3.89E-06 | Female |  |
| 271 | 2.55E-05 | Female |  | 3.90E-06 | Female |  |
| 272 | 2.79E-05 | Female |  | 3.90E-06 | Female |  |
| 273 | 3.19E-05 | Female |  | 3.90E-06 | Female |  |
| 274 | 2.73E-05 | Female |  | 3.91E-06 | Female |  |
| 275 | 2.91E-05 | Female |  | 3.92E-06 | Female |  |
| 276 | 2.72E-05 | Female |  | 3.92E-06 | Female |  |
| 277 | 2.34E-05 | Female |  | 3.92E-06 | Female |  |
| 278 | 2.82E-05 | Female |  | 3.96E-06 | Female |  |
| 279 | 3.31E-05 | Female |  | 3.96E-06 | Female |  |
| 280 | 2.54E-05 | Female |  | 3.96E-06 | Female |  |
| 281 | 2.85E-05 | Female |  | 3.97E-06 | Female |  |
| 282 | 2.50E-05 | Female |  | 3.98E-06 | Female |  |
| 283 | 2.75E-05 | Female |  | 3.98E-06 | Female |  |
| 284 | 2.80E-05 | Female |  | 3.99E-06 | Female |  |
| 285 | 2.64E-05 | Female |  | 3.99E-06 | Female |  |
| 286 | 2.47E-05 | Female |  | 4.00E-06 | Female |  |
| 287 | 3.40E-05 | Female |  | 4.02E-06 | Female |  |
| 288 | 3.00E-05 | Female |  | 4.05E-06 | Female |  |
| 289 | 2.90E-05 | Female |  | 4.06E-06 | Female |  |
| 290 | 2.69E-05 | Female |  | 4.06E-06 | Female |  |
| 291 | 2.56E-05 | Female |  | 4.06E-06 | Female |  |
| 292 | 2.59E-05 | Female |  | 4.07E-06 | Female |  |
| 293 | 2.70E-05 | Female |  | 4.07E-06 | Female |  |
| 294 | 2.70E-05 | Female |  | 4.07E-06 | Female |  |
| 295 | 2.78E-05 | Female |  | 4.09E-06 | Female |  |
| 296 | 2.83E-05 | Female |  | 4.09E-06 | Female |  |
| 297 | 3.12E-05 | Female |  | 4.09E-06 | Female |  |
| 298 | 2.97E-05 | Female |  | 4.10E-06 | Female |  |
| 299 | 3.21E-05 | Female |  | 4.10E-06 | Female |  |
| 300 | 2.53E-05 | Female |  | 4.10E-06 | Female |  |
| 301 | 3.27E-05 | Female |  | 4.10E-06 | Female |  |
| 302 | 3.18E-05 | Female |  | 4.12E-06 | Female |  |
| 303 | 2.56E-05 | Female |  | 4.12E-06 | Female |  |
| 304 | 2.88E-05 | Female |  | 4.13E-06 | Female |  |
| 305 | 2.67E-05 | Female |  | 4.14E-06 | Female |  |
| 306 | 2.98E-05 | Female |  | 4.15E-06 | Female |  |
| 307 | 3.19E-05 | Female |  | 4.15E-06 | Female |  |
| 308 | 3.08E-05 | Female |  | 4.15E-06 | Female |  |
| 309 | 2.92E-05 | Female |  | 4.18E-06 | Female |  |
| 310 | 3.07E-05 | Female |  | 4.18E-06 | Female |  |
| 311 | 3.04E-05 | Female |  | 4.19E-06 | Female |  |
| 312 | 2.79E-05 | Female |  | 4.20E-06 | Female |  |
| 313 | 2.79E-05 | Female |  | 4.20E-06 | Female |  |
| 314 | 3.18E-05 | Female |  | 4.20E-06 | Female |  |
| 315 | 2.72E-05 | Female |  | 4.20E-06 | Female |  |
| 316 | 3.24E-05 | Female |  | 4.20E-06 | Female |  |
| 317 | 2.54E-05 | Female |  | 4.21E-06 | Female |  |
| 318 | 3.06E-05 | Female |  | 4.22E-06 | Female |  |
| 319 | 2.76E-05 | Female |  | 4.22E-06 | Female |  |
| 320 | 3.04E-05 | Female |  | 4.23E-06 | Female |  |
| 321 | 2.66E-05 | Female |  | 4.25E-06 | Female |  |
| 322 | 2.57E-05 | Female |  | 4.26E-06 | Female |  |
| 323 | 2.83E-05 | Female |  | 4.26E-06 | Female |  |
| 324 | 2.51E-05 | Female |  | 4.27E-06 | Female |  |
| 325 | 2.67E-05 | Female |  | 4.28E-06 | Female |  |
| 326 | 2.96E-05 | Female |  | 4.29E-06 | Female |  |
| 327 | 2.62E-05 | Female |  | 4.30E-06 | Female |  |
| 328 | 2.91E-05 | Female |  | 4.32E-06 | Female |  |
| 329 | 2.75E-05 | Female |  | 4.33E-06 | Female |  |
| 330 | 2.71E-05 | Female |  | 4.33E-06 | Female |  |
| 331 | 2.96E-05 | Female |  | 4.34E-06 | Female |  |
| 332 | 2.96E-05 | Female |  | 4.35E-06 | Female |  |
| 333 | 3.12E-05 | Female |  | 4.35E-06 | Female |  |
| 334 | 2.77E-05 | Female |  | 4.35E-06 | Female |  |
| 335 | 3.10E-05 | Female |  | 4.37E-06 | Female |  |
| 336 | 3.03E-05 | Female |  | 4.38E-06 | Female |  |
| 337 | 2.82E-05 | Female |  | 4.38E-06 | Female |  |
| 338 | 3.24E-05 | Female |  | 4.39E-06 | Female |  |
| 339 | 2.99E-05 | Female |  | 4.40E-06 | Female |  |
| 340 | 2.61E-05 | Female |  | 4.41E-06 | Female |  |
| 341 | 2.67E-05 | Female |  | 4.42E-06 | Female |  |
| 342 | 2.41E-05 | Female |  | 4.42E-06 | Female |  |
| 343 | 2.82E-05 | Female |  | 4.42E-06 | Female |  |
| 344 | 2.76E-05 | Female |  | 4.43E-06 | Female |  |
| 345 | 2.93E-05 | Female |  | 4.43E-06 | Female |  |
| 346 | 2.84E-05 | Female |  | 4.43E-06 | Female |  |
| 347 | 2.74E-05 | Female |  | 4.43E-06 | Female |  |
| 348 | 2.67E-05 | Female |  | 4.44E-06 | Female |  |
| 349 | 2.95E-05 | Female |  | 4.46E-06 | Female |  |
| 350 | 2.91E-05 | Female |  | 4.47E-06 | Female |  |
| 351 | 3.30E-05 | Female |  | 4.48E-06 | Female |  |
| 352 | 3.22E-05 | Female |  | 4.48E-06 | Female |  |
| 353 | 3.20E-05 | Female |  | 4.50E-06 | Female |  |
| 354 | 3.12E-05 | Female |  | 4.51E-06 | Female |  |
| 355 | 2.75E-05 | Female |  | 4.52E-06 | Female |  |
| 356 | 2.32E-05 | Female |  | 4.53E-06 | Female |  |
| 357 | 2.98E-05 | Female |  | 4.53E-06 | Female |  |
| 358 | 2.56E-05 | Female |  | 4.55E-06 | Female |  |
| 359 | 2.75E-05 | Female |  | 4.55E-06 | Female |  |
| 360 | 1.95E-05 | Female |  | 4.55E-06 | Female |  |
| 361 | 2.99E-05 | Female |  | 4.55E-06 | Female |  |
| 362 | 3.22E-05 | Female |  | 4.56E-06 | Female |  |
| 363 | 3.20E-05 | Female |  | 4.56E-06 | Female |  |
| 364 | 2.99E-05 | Female |  | 4.57E-06 | Female |  |
| 365 | 2.67E-05 | Female |  | 4.57E-06 | Female |  |
| 366 | 2.53E-05 | Female |  | 4.58E-06 | Female |  |
| 367 | 2.92E-05 | Female |  | 4.59E-06 | Female |  |
| 368 | 3.06E-05 | Female |  | 4.60E-06 | Female |  |
| 369 | 2.90E-05 | Female |  | 4.60E-06 | Female |  |
| 370 | 2.68E-05 | Female |  | 4.61E-06 | Female |  |
| 371 | 2.88E-05 | Female |  | 4.63E-06 | Female |  |
| 372 | 2.58E-05 | Female |  | 4.64E-06 | Female |  |
| 373 | 2.97E-05 | Female |  | 4.64E-06 | Female |  |
| 374 | 3.01E-05 | Female |  | 4.67E-06 | Female |  |
| 375 | 2.60E-05 | Female |  | 4.68E-06 | Female |  |
| 376 | 3.14E-05 | Female |  | 4.69E-06 | Female |  |
| 377 | 2.74E-05 | Female |  | 4.71E-06 | Female |  |
| 378 | 3.04E-05 | Female |  | 4.71E-06 | Female |  |
| 379 | 2.86E-05 | Female |  | 4.71E-06 | Female |  |
| 380 | 3.42E-05 | Female |  | 4.72E-06 | Female |  |
| 381 | 2.57E-05 | Female |  | 4.73E-06 | Female |  |
| 382 | 3.01E-05 | Female |  | 4.73E-06 | Female |  |
| 383 | 2.69E-05 | Female |  | 4.74E-06 | Female |  |
| 384 | 3.31E-05 | Female |  | 4.74E-06 | Female |  |
| 385 | 3.16E-05 | Female |  | 4.74E-06 | Female |  |
| 386 | 2.77E-05 | Female |  | 4.74E-06 | Female |  |
| 387 | 2.97E-05 | Female |  | 4.77E-06 | Female |  |
| 388 | 2.90E-05 | Female |  | 4.77E-06 | Female |  |
| 389 | 2.50E-05 | Female |  | 4.78E-06 | Female |  |
| 390 | 3.03E-05 | Female |  | 4.78E-06 | Female |  |
| 391 | 2.52E-05 | Female |  | 4.78E-06 | Female |  |
| 392 | 2.98E-05 | Female |  | 4.79E-06 | Female |  |
| 393 | 3.26E-05 | Female |  | 4.80E-06 | Female |  |
| 394 | 3.02E-05 | Female |  | 4.80E-06 | Female |  |
| 395 | 2.69E-05 | Female |  | 4.80E-06 | Female |  |
| 396 | 2.86E-05 | Female |  | 4.80E-06 | Female |  |
| 397 | 3.01E-05 | Female |  | 4.83E-06 | Female |  |
| 398 | 2.62E-05 | Female |  | 4.83E-06 | Female |  |
| 399 | 2.83E-05 | Female |  | 4.84E-06 | Female |  |
| 400 | 3.27E-05 | Female |  | 4.85E-06 | Female |  |
| 401 | 3.01E-05 | Female |  | 4.85E-06 | Female |  |
| 402 | 2.67E-05 | Female |  | 4.86E-06 | Female |  |
| 403 | 2.80E-05 | Female |  | 4.86E-06 | Female |  |
| 404 | 3.11E-05 | Female |  | 4.86E-06 | Female |  |
| 405 | 2.84E-05 | Female |  | 4.89E-06 | Female |  |
| 406 | 2.95E-05 | Female |  | 4.89E-06 | Female |  |
| 407 | 3.03E-05 | Female |  | 4.90E-06 | Female |  |
| 408 | 2.52E-05 | Female |  | 4.90E-06 | Female |  |
| 409 | 2.49E-05 | Female |  | 4.91E-06 | Female |  |
| 410 | 2.88E-05 | Female |  | 4.92E-06 | Female |  |
| 411 | 3.03E-05 | Female |  | 4.94E-06 | Female |  |
| 412 | 2.96E-05 | Female |  | 4.94E-06 | Female |  |
| 413 | 2.80E-05 | Female |  | 4.95E-06 | Female |  |
| 414 | 2.83E-05 | Female |  | 4.96E-06 | Female |  |
| 415 | 3.37E-05 | Female |  | 4.96E-06 | Female |  |
| 416 | 2.84E-05 | Female |  | 4.96E-06 | Female |  |
| 417 | 2.94E-05 | Female |  | 4.96E-06 | Female |  |
| 418 | 3.06E-05 | Female |  | 4.97E-06 | Female |  |
| 419 | 3.15E-05 | Female |  | 4.97E-06 | Female |  |
| 420 | 3.05E-05 | Female |  | 4.97E-06 | Female |  |
| 421 | 2.85E-05 | Female |  | 4.98E-06 | Female |  |
| 422 | 2.83E-05 | Female |  | 4.99E-06 | Female |  |
| 423 | 3.10E-05 | Female |  | 4.99E-06 | Female |  |
| 424 | 2.92E-05 | Female |  | 5.00E-06 | Female |  |
| 425 | 2.53E-05 | Female |  | 5.00E-06 | Female |  |
| 426 | 2.88E-05 | Female |  | 5.02E-06 | Female |  |
| 427 | 3.18E-05 | Female |  | 5.03E-06 | Female |  |
| 428 | 2.84E-05 | Female |  | 5.04E-06 | Female |  |
| 429 | 3.09E-05 | Female |  | 5.05E-06 | Female |  |
| 430 | 3.50E-05 | Female |  | 5.07E-06 | Female |  |
| 431 | 2.84E-05 | Female |  | 5.07E-06 | Female |  |
| 432 | 2.65E-05 | Female |  | 5.08E-06 | Female |  |
| 433 | 3.02E-05 | Female |  | 5.09E-06 | Female |  |
| 434 | 2.85E-05 | Female |  | 5.09E-06 | Female |  |
| 435 | 2.98E-05 | Female |  | 5.09E-06 | Female |  |
| 436 | 2.78E-05 | Female |  | 5.10E-06 | Female |  |
| 437 | 3.04E-05 | Female |  | 5.10E-06 | Female |  |
| 438 | 2.86E-05 | Female |  | 5.17E-06 | Female |  |
| 439 | 3.10E-05 | Female |  | 5.17E-06 | Female |  |
| 440 | 3.18E-05 | Female |  | 5.17E-06 | Female |  |
| 441 | 3.06E-05 | Female |  | 5.18E-06 | Female |  |
| 442 | 3.29E-05 | Female |  | 5.19E-06 | Female |  |
| 443 | 2.93E-05 | Female |  | 5.20E-06 | Female |  |
| 444 | 3.29E-05 | Female |  | 5.20E-06 | Female |  |
| 445 | 2.99E-05 | Female |  | 5.23E-06 | Female |  |
| 446 | 3.09E-05 | Female |  | 5.24E-06 | Female |  |
| 447 | 3.02E-05 | Female |  | 5.24E-06 | Female |  |
| 448 | 2.73E-05 | Female |  | 5.24E-06 | Female |  |
| 449 | 2.68E-05 | Female |  | 5.25E-06 | Female |  |
| 450 | 3.24E-05 | Female |  | 5.25E-06 | Female |  |
| 451 | 2.73E-05 | Female |  | 5.25E-06 | Female |  |
| 452 | 3.29E-05 | Female |  | 5.27E-06 | Female |  |
| 453 | 2.48E-05 | Female |  | 5.27E-06 | Female |  |
| 454 | 2.87E-05 | Female |  | 5.27E-06 | Female |  |
| 455 | 2.62E-05 | Female |  | 5.28E-06 | Female |  |
| 456 | 2.95E-05 | Female |  | 5.29E-06 | Female |  |
| 457 | 2.47E-05 | Female |  | 5.30E-06 | Female |  |
| 458 | 3.10E-05 | Female |  | 5.32E-06 | Female |  |
| 459 | 2.87E-05 | Female |  | 5.33E-06 | Female |  |
| 460 | 2.84E-05 | Female |  | 5.34E-06 | Female |  |
| 461 | 3.15E-05 | Female |  | 5.34E-06 | Female |  |
| 462 | 3.47E-05 | Female |  | 5.34E-06 | Female |  |
| 463 | 2.86E-05 | Female |  | 5.34E-06 | Female |  |
| 464 | 3.44E-05 | Female |  | 5.38E-06 | Female |  |
| 465 | 3.21E-05 | Female |  | 5.40E-06 | Female |  |
| 466 | 2.54E-05 | Female |  | 5.40E-06 | Female |  |
| 467 | 2.84E-05 | Female |  | 5.41E-06 | Female |  |
| 468 | 2.82E-05 | Female |  | 5.41E-06 | Female |  |
| 469 | 2.81E-05 | Female |  | 5.41E-06 | Female |  |
| 470 | 3.20E-05 | Female |  | 5.42E-06 | Female |  |
| 471 | 2.96E-05 | Female |  | 5.43E-06 | Female |  |
| 472 | 3.15E-05 | Female |  | 5.44E-06 | Female |  |
| 473 | 2.93E-05 | Female |  | 5.44E-06 | Female |  |
| 474 | 2.88E-05 | Female |  | 5.46E-06 | Female |  |
| 475 | 3.42E-05 | Female |  | 5.47E-06 | Female |  |
| 476 | 3.53E-05 | Female |  | 5.47E-06 | Female |  |
| 477 | 2.83E-05 | Female |  | 5.47E-06 | Female |  |
| 478 | 3.04E-05 | Female |  | 5.48E-06 | Female |  |
| 479 | 3.02E-05 | Female |  | 5.49E-06 | Female |  |
| 480 | 3.16E-05 | Female |  | 5.49E-06 | Female |  |
| 481 | 3.58E-05 | Female |  | 5.51E-06 | Female |  |
| 482 | 3.54E-05 | Female |  | 5.52E-06 | Female |  |
| 483 | 3.31E-05 | Female |  | 5.53E-06 | Female |  |
| 484 | 3.54E-05 | Female |  | 5.55E-06 | Female |  |
| 485 | 2.74E-05 | Female |  | 5.55E-06 | Female |  |
| 486 | 2.79E-05 | Female |  | 5.57E-06 | Female |  |
| 487 | 2.46E-05 | Female |  | 5.59E-06 | Female |  |
| 488 | 2.64E-05 | Female |  | 5.61E-06 | Female |  |
| 489 | 2.95E-05 | Female |  | 5.62E-06 | Female |  |
| 490 | 3.00E-05 | Female |  | 5.62E-06 | Female |  |
| 491 | 3.07E-05 | Female |  | 5.62E-06 | Female |  |
| 492 | 2.86E-05 | Female |  | 5.65E-06 | Female |  |
| 493 | 2.77E-05 | Female |  | 5.66E-06 | Female |  |
| 494 | 3.61E-05 | Female |  | 5.66E-06 | Female |  |
| 495 | 3.28E-05 | Female |  | 5.67E-06 | Female |  |
| 496 | 2.81E-05 | Female |  | 5.68E-06 | Female |  |
| 497 | 3.18E-05 | Female |  | 5.68E-06 | Female |  |
| 498 | 2.85E-05 | Female |  | 5.68E-06 | Female |  |
| 499 | 2.63E-05 | Female |  | 5.70E-06 | Female |  |
| 500 | 3.07E-05 | Female |  | 5.73E-06 | Female |  |
| 501 | 3.47E-05 | Female |  | 5.74E-06 | Female |  |
| 502 | 3.02E-05 | Female |  | 5.74E-06 | Female |  |
| 503 | 2.97E-05 | Female |  | 5.77E-06 | Female |  |
| 504 | 3.08E-05 | Female |  | 5.79E-06 | Female |  |
| 505 | 3.10E-05 | Female |  | 5.80E-06 | Female |  |
| 506 | 3.24E-05 | Female |  | 5.82E-06 | Female |  |
| 507 | 2.85E-05 | Female |  | 5.86E-06 | Female |  |
| 508 | 2.83E-05 | Female |  | 5.87E-06 | Female |  |
| 509 | 3.08E-05 | Female |  | 5.90E-06 | Female |  |
| 510 | 3.23E-05 | Female |  | 5.91E-06 | Female |  |
| 511 | 2.40E-05 | Female |  | 5.92E-06 | Female |  |
| 512 | 2.69E-05 | Female |  | 5.94E-06 | Female |  |
| 513 | 2.85E-05 | Female |  | 5.94E-06 | Female |  |
| 514 | 2.82E-05 | Female |  | 5.95E-06 | Female |  |
| 515 | 2.79E-05 | Female |  | 5.97E-06 | Female |  |
| 516 | 3.09E-05 | Female |  | 6.00E-06 | Female |  |
| 517 | 3.56E-05 | Female |  | 6.03E-06 | Female |  |
| 518 | 3.21E-05 | Female |  | 6.03E-06 | Female |  |
| 519 | 2.95E-05 | Female |  | 6.03E-06 | Female |  |
| 520 | 3.07E-05 | Female |  | 6.04E-06 | Female |  |
| 521 | 3.21E-05 | Female |  | 6.04E-06 | Female |  |
| 522 | 3.24E-05 | Female |  | 6.04E-06 | Female |  |
| 523 | 3.20E-05 | Female |  | 6.05E-06 | Female |  |
| 524 | 2.54E-05 | Female |  | 6.07E-06 | Female |  |
| 525 | 3.30E-05 | Female |  | 6.09E-06 | Female |  |
| 526 | 2.87E-05 | Female |  | 6.09E-06 | Female |  |
| 527 | 2.53E-05 | Female |  | 6.10E-06 | Female |  |
| 528 | 2.90E-05 | Female |  | 6.10E-06 | Female |  |
| 529 | 2.82E-05 | Female |  | 6.14E-06 | Female |  |
| 530 | 3.14E-05 | Female |  | 6.15E-06 | Female |  |
| 531 | 3.08E-05 | Female |  | 6.16E-06 | Female |  |
| 532 | 2.92E-05 | Female |  | 6.17E-06 | Female |  |
| 533 | 3.33E-05 | Female |  | 6.18E-06 | Female |  |
| 534 | 3.23E-05 | Female |  | 6.19E-06 | Female |  |
| 535 | 2.63E-05 | Female |  | 6.20E-06 | Female |  |
| 536 | 2.93E-05 | Female |  | 6.21E-06 | Female |  |
| 537 | 3.13E-05 | Female |  | 6.22E-06 | Female |  |
| 538 | 2.74E-05 | Female |  | 6.23E-06 | Female |  |
| 539 | 3.07E-05 | Female |  | 6.27E-06 | Female |  |
| 540 | 3.01E-05 | Female |  | 6.28E-06 | Female |  |
| 541 | 3.25E-05 | Female |  | 6.28E-06 | Female |  |
| 542 | 3.43E-05 | Female |  | 6.30E-06 | Female |  |
| 543 | 3.15E-05 | Female |  | 6.30E-06 | Female |  |
| 544 | 3.48E-05 | Female |  | 6.30E-06 | Female |  |
| 545 | 3.29E-05 | Female |  | 6.31E-06 | Female |  |
| 546 | 3.17E-05 | Female |  | 6.31E-06 | Female |  |
| 547 | 3.38E-05 | Female |  | 6.32E-06 | Female |  |
| 548 | 2.82E-05 | Female |  | 6.32E-06 | Female |  |
| 549 | 3.00E-05 | Female |  | 6.33E-06 | Female |  |
| 550 | 3.24E-05 | Female |  | 6.37E-06 | Female |  |
| 551 | 2.73E-05 | Female |  | 6.37E-06 | Female |  |
| 552 | 3.63E-05 | Female |  | 6.38E-06 | Female |  |
| 553 | 3.12E-05 | Female |  | 6.39E-06 | Female |  |
| 554 | 2.63E-05 | Female |  | 6.42E-06 | Female |  |
| 555 | 3.01E-05 | Female |  | 6.42E-06 | Female |  |
| 556 | 2.58E-05 | Female |  | 6.45E-06 | Female |  |
| 557 | 3.02E-05 | Female |  | 6.47E-06 | Female |  |
| 558 | 2.72E-05 | Female |  | 6.47E-06 | Female |  |
| 559 | 3.58E-05 | Female |  | 6.48E-06 | Female |  |
| 560 | 3.20E-05 | Female |  | 6.49E-06 | Female |  |
| 561 | 2.94E-05 | Female |  | 6.50E-06 | Female |  |
| 562 | 3.11E-05 | Female |  | 6.51E-06 | Female |  |
| 563 | 2.92E-05 | Female |  | 6.55E-06 | Female |  |
| 564 | 2.85E-05 | Female |  | 6.56E-06 | Female |  |
| 565 | 2.95E-05 | Female |  | 6.59E-06 | Female |  |
| 566 | 3.13E-05 | Female |  | 6.59E-06 | Female |  |
| 567 | 3.07E-05 | Female |  | 6.62E-06 | Female |  |
| 568 | 3.27E-05 | Female |  | 6.64E-06 | Female |  |
| 569 | 2.94E-05 | Female |  | 6.66E-06 | Female |  |
| 570 | 2.67E-05 | Female |  | 6.67E-06 | Female |  |
| 571 | 3.67E-05 | Female |  | 6.69E-06 | Female |  |
| 572 | 3.42E-05 | Female |  | 6.70E-06 | Female |  |
| 573 | 3.13E-05 | Female |  | 6.71E-06 | Female |  |
| 574 | 3.08E-05 | Female |  | 6.73E-06 | Female |  |
| 575 | 3.09E-05 | Female |  | 6.73E-06 | Female |  |
| 576 | 3.18E-05 | Female |  | 6.74E-06 | Female |  |
| 577 | 3.16E-05 | Female |  | 6.75E-06 | Female |  |
| 578 | 3.18E-05 | Female |  | 6.75E-06 | Female |  |
| 579 | 3.05E-05 | Female |  | 6.76E-06 | Female |  |
| 580 | 3.56E-05 | Female |  | 6.76E-06 | Female |  |
| 581 | 3.12E-05 | Female |  | 6.76E-06 | Female |  |
| 582 | 3.14E-05 | Female |  | 6.76E-06 | Female |  |
| 583 | 2.96E-05 | Female |  | 6.78E-06 | Female |  |
| 584 | 3.08E-05 | Female |  | 6.79E-06 | Female |  |
| 585 | 3.16E-05 | Female |  | 6.80E-06 | Female |  |
| 586 | 3.18E-05 | Female |  | 6.82E-06 | Female |  |
| 587 | 2.74E-05 | Female |  | 6.83E-06 | Female |  |
| 588 | 3.54E-05 | Female |  | 6.83E-06 | Female |  |
| 589 | 3.13E-05 | Female |  | 6.85E-06 | Female |  |
| 590 | 2.90E-05 | Female |  | 6.86E-06 | Female |  |
| 591 | 3.02E-05 | Female |  | 6.87E-06 | Female |  |
| 592 | 3.02E-05 | Female |  | 6.90E-06 | Female |  |
| 593 | 3.13E-05 | Female |  | 6.90E-06 | Female |  |
| 594 | 3.23E-05 | Female |  | 6.93E-06 | Female |  |
| 595 | 3.44E-05 | Female |  | 6.93E-06 | Female |  |
| 596 | 3.10E-05 | Female |  | 6.95E-06 | Female |  |
| 597 | 3.40E-05 | Female |  | 6.96E-06 | Female |  |
| 598 | 3.14E-05 | Female |  | 6.97E-06 | Female |  |
| 599 | 2.81E-05 | Female |  | 6.98E-06 | Female |  |
| 600 | 3.18E-05 | Female |  | 6.98E-06 | Female |  |
| 601 | 3.35E-05 | Female |  | 7.07E-06 | Female |  |
| 602 | 3.42E-05 | Female |  | 7.07E-06 | Female |  |
| 603 | 3.38E-05 | Female |  | 7.09E-06 | Female |  |
| 604 | 3.29E-05 | Female |  | 7.09E-06 | Female |  |
| 605 | 2.93E-05 | Female |  | 7.10E-06 | Female |  |
| 606 | 2.79E-05 | Female |  | 7.10E-06 | Female |  |
| 607 | 2.88E-05 | Female |  | 7.10E-06 | Female |  |
| 608 | 3.36E-05 | Female |  | 7.12E-06 | Female |  |
| 609 | 2.89E-05 | Female |  | 7.16E-06 | Female |  |
| 610 | 3.04E-05 | Female |  | 7.22E-06 | Female |  |
| 611 | 3.25E-05 | Female |  | 7.29E-06 | Female |  |
| 612 | 2.72E-05 | Female |  | 7.29E-06 | Female |  |
| 613 | 3.39E-05 | Female |  | 7.30E-06 | Female |  |
| 614 | 2.95E-05 | Female |  | 7.31E-06 | Female |  |
| 615 | 3.02E-05 | Female |  | 7.31E-06 | Female |  |
| 616 | 3.12E-05 | Female |  | 7.39E-06 | Female |  |
| 617 | 3.16E-05 | Female |  | 7.41E-06 | Female |  |
| 618 | 2.74E-05 | Female |  | 7.46E-06 | Female |  |
| 619 | 3.51E-05 | Female |  | 7.47E-06 | Female |  |
| 620 | 3.17E-05 | Female |  | 7.50E-06 | Female |  |
| 621 | 3.35E-05 | Female |  | 7.50E-06 | Female |  |
| 622 | 3.17E-05 | Female |  | 7.53E-06 | Female |  |
| 623 | 3.29E-05 | Female |  | 7.57E-06 | Female |  |
| 624 | 3.53E-05 | Female |  | 7.57E-06 | Female |  |
| 625 | 3.54E-05 | Female |  | 7.59E-06 | Female |  |
| 626 | 3.12E-05 | Female |  | 7.63E-06 | Female |  |
| 627 | 2.82E-05 | Female |  | 7.65E-06 | Female |  |
| 628 | 3.57E-05 | Female |  | 7.69E-06 | Female |  |
| 629 | 3.12E-05 | Female |  | 7.75E-06 | Female |  |
| 630 | 3.54E-05 | Female |  | 7.82E-06 | Female |  |
| 631 | 2.80E-05 | Female |  | 7.84E-06 | Female |  |
| 632 | 2.97E-05 | Female |  | 7.87E-06 | Female |  |
| 633 | 2.94E-05 | Female |  | 7.89E-06 | Female |  |
| 634 | 3.40E-05 | Female |  | 7.90E-06 | Female |  |
| 635 | 3.39E-05 | Female |  | 7.91E-06 | Female |  |
| 636 | 2.98E-05 | Female |  | 7.97E-06 | Female |  |
| 637 | 3.92E-05 | Female |  | 7.99E-06 | Female |  |
| 638 | 3.14E-05 | Female |  | 7.99E-06 | Female |  |
| 639 | 3.09E-05 | Female |  | 7.99E-06 | Female |  |
| 640 | 3.34E-05 | Female |  | 8.06E-06 | Female |  |
| 641 | 3.59E-05 | Female |  | 8.08E-06 | Female |  |
| 642 | 3.28E-05 | Female |  | 8.08E-06 | Female |  |
| 643 | 3.50E-05 | Female |  | 8.10E-06 | Female |  |
| 644 | 3.69E-05 | Female |  | 8.11E-06 | Female |  |
| 645 | 3.46E-05 | Female |  | 8.13E-06 | Female |  |
| 646 | 3.49E-05 | Female |  | 8.16E-06 | Female |  |
| 647 | 3.13E-05 | Female |  | 8.16E-06 | Female |  |
| 648 | 3.08E-05 | Female |  | 8.17E-06 | Female |  |
| 649 | 3.35E-05 | Female |  | 8.19E-06 | Female |  |
| 650 | 3.64E-05 | Female |  | 8.21E-06 | Female |  |
| 651 | 3.66E-05 | Female |  | 8.22E-06 | Female |  |
| 652 | 3.39E-05 | Female |  | 8.23E-06 | Female |  |
| 653 | 3.37E-05 | Female |  | 8.24E-06 | Female |  |
| 654 | 3.53E-05 | Female |  | 8.26E-06 | Female |  |
| 655 | 2.93E-05 | Female |  | 8.30E-06 | Female |  |
| 656 | 3.85E-05 | Female |  | 8.37E-06 | Female |  |
| 657 | 3.32E-05 | Female |  | 8.42E-06 | Female |  |
| 658 | 3.39E-05 | Female |  | 8.44E-06 | Female |  |
| 659 | 2.83E-05 | Female |  | 8.44E-06 | Female |  |
| 660 | 3.23E-05 | Female |  | 8.44E-06 | Female |  |
| 661 | 2.94E-05 | Female |  | 8.47E-06 | Female |  |
| 662 | 2.98E-05 | Female |  | 8.48E-06 | Female |  |
| 663 | 3.38E-05 | Female |  | 8.48E-06 | Female |  |
| 664 | 3.32E-05 | Female |  | 8.51E-06 | Female |  |
| 665 | 3.33E-05 | Female |  | 8.52E-06 | Female |  |
| 666 | 3.27E-05 | Female |  | 8.57E-06 | Female |  |
| 667 | 3.17E-05 | Female |  | 8.57E-06 | Female |  |
| 668 | 3.25E-05 | Female |  | 8.59E-06 | Female |  |
| 669 | 2.93E-05 | Female |  | 8.61E-06 | Female |  |
| 670 | 3.68E-05 | Female |  | 8.61E-06 | Female |  |
| 671 | 3.28E-05 | Female |  | 8.64E-06 | Female |  |
| 672 | 3.57E-05 | Female |  | 8.72E-06 | Female |  |
| 673 | 3.34E-05 | Female |  | 8.81E-06 | Female |  |
| 674 | 3.14E-05 | Female |  | 8.84E-06 | Female |  |
| 675 | 3.58E-05 | Female |  | 8.87E-06 | Female |  |
| 676 | 3.42E-05 | Female |  | 8.90E-06 | Female |  |
| 677 | 3.57E-05 | Female |  | 8.93E-06 | Female |  |
| 678 | 3.86E-05 | Female |  | 8.98E-06 | Female |  |
| 679 | 3.66E-05 | Female |  | 8.99E-06 | Female |  |
| 680 | 3.88E-05 | Female |  | 9.03E-06 | Female |  |
| 681 | 3.40E-05 | Female |  | 9.08E-06 | Female |  |
| 682 | 3.41E-05 | Female |  | 9.17E-06 | Female |  |
| 683 | 3.78E-05 | Female |  | 9.23E-06 | Female |  |
| 684 | 3.52E-05 | Female |  | 9.24E-06 | Female |  |
| 685 | 3.72E-05 | Female |  | 9.25E-06 | Female |  |
| 686 | 2.98E-05 | Female |  | 9.28E-06 | Female |  |
| 687 | 3.05E-05 | Female |  | 9.28E-06 | Female |  |
| 688 | 3.29E-05 | Female |  | 9.30E-06 | Female |  |
| 689 | 3.24E-05 | Female |  | 9.31E-06 | Female |  |
| 690 | 3.25E-05 | Female |  | 9.36E-06 | Female |  |
| 691 | 3.52E-05 | Female |  | 9.36E-06 | Female |  |
| 692 | 3.37E-05 | Female |  | 9.37E-06 | Female |  |
| 693 | 3.37E-05 | Female |  | 9.40E-06 | Female |  |
| 694 | 3.24E-05 | Female |  | 9.48E-06 | Female |  |
| 695 | 3.24E-05 | Female |  | 9.52E-06 | Female |  |
| 696 | 3.32E-05 | Female |  | 9.53E-06 | Female |  |
| 697 | 4.02E-05 | Female |  | 9.54E-06 | Female |  |
| 698 | 3.31E-05 | Female |  | 9.60E-06 | Female |  |
| 699 | 3.67E-05 | Female |  | 9.63E-06 | Female |  |
| 700 | 3.95E-05 | Female |  | 9.84E-06 | Female |  |
| 701 | 3.52E-05 | Female |  | 9.87E-06 | Female |  |
| 702 | 3.70E-05 | Female |  | 9.90E-06 | Female |  |
| 703 | 3.43E-05 | Female |  | 1.01E-05 | Female |  |
| 704 | 3.56E-05 | Female |  | 1.01E-05 | Female |  |
| 705 | 3.39E-05 | Female |  | 1.02E-05 | Female |  |
| 706 | 3.39E-05 | Female |  | 1.02E-05 | Female |  |
| 707 | 3.65E-05 | Female |  | 1.02E-05 | Female |  |
| 708 | 3.30E-05 | Female |  | 1.03E-05 | Female |  |
| 709 | 3.35E-05 | Female |  | 1.04E-05 | Female |  |
| 710 | 3.47E-05 | Female |  | 1.04E-05 | Female |  |
| 711 | 4.24E-05 | Female |  | 1.04E-05 | Female |  |
| 712 | 3.66E-05 | Female |  | 1.04E-05 | Female |  |
| 713 | 3.19E-05 | Female |  | 1.04E-05 | Female |  |
| 714 | 3.91E-05 | Female |  | 1.04E-05 | Female |  |
| 715 | 3.74E-05 | Female |  | 1.06E-05 | Female |  |
| 716 | 3.24E-05 | Female |  | 1.06E-05 | Female |  |
| 717 | 3.94E-05 | Female |  | 1.07E-05 | Female |  |
| 718 | 2.95E-05 | Female |  | 1.07E-05 | Female |  |
| 719 | 3.65E-05 | Female |  | 1.07E-05 | Female |  |
| 720 | 3.51E-05 | Female |  | 1.07E-05 | Female |  |
| 721 | 3.93E-05 | Female |  | 1.09E-05 | Female |  |
| 722 | 3.88E-05 | Female |  | 1.09E-05 | Female |  |
| 723 | 3.51E-05 | Female |  | 1.10E-05 | Female |  |
| 724 | 3.90E-05 | Female |  | 1.10E-05 | Female |  |
| 725 | 4.18E-05 | Female |  | 1.11E-05 | Female |  |
| 726 | 3.00E-05 | Female |  | 1.11E-05 | Female |  |
| 727 | 3.51E-05 | Female |  | 1.11E-05 | Female |  |
| 728 | 3.70E-05 | Female |  | 1.12E-05 | Female |  |
| 729 | 2.92E-05 | Female |  | 1.12E-05 | Female |  |
| 730 | 3.80E-05 | Female |  | 1.14E-05 | Female |  |
| 731 | 3.63E-05 | Female |  | 1.14E-05 | Female |  |
| 732 | 3.86E-05 | Female |  | 1.15E-05 | Female |  |
| 733 | 3.73E-05 | Female |  | 1.18E-05 | Female |  |
| 734 | 3.36E-05 | Female |  | 1.18E-05 | Female |  |
| 735 | 3.92E-05 | Female |  | 1.20E-05 | Female |  |
| 736 | 3.88E-05 | Female |  | 1.21E-05 | Female |  |
| 737 | 3.21E-05 | Female |  | 1.21E-05 | Female |  |
| 738 | 3.53E-05 | Female |  | 1.21E-05 | Female |  |
| 739 | 3.70E-05 | Female |  | 1.21E-05 | Female |  |
| 740 | 3.92E-05 | Female |  | 1.23E-05 | Female |  |
| 741 | 3.83E-05 | Female |  | 1.24E-05 | Female |  |
| 742 | 3.38E-05 | Female |  | 1.24E-05 | Female |  |
| 743 | 3.30E-05 | Female |  | 1.25E-05 | Female |  |
| 744 | 3.98E-05 | Female |  | 1.25E-05 | Female |  |
| 745 | 3.46E-05 | Female |  | 1.26E-05 | Female |  |
| 746 | 3.99E-05 | Female |  | 1.26E-05 | Female |  |
| 747 | 3.71E-05 | Female |  | 1.26E-05 | Female |  |
| 748 | 3.85E-05 | Female |  | 1.27E-05 | Female |  |
| 749 | 3.75E-05 | Female |  | 1.28E-05 | Female |  |
| 750 | 3.28E-05 | Female |  | 1.29E-05 | Female |  |
| 751 | 3.76E-05 | Female |  | 1.29E-05 | Female |  |
| 752 | 3.82E-05 | Female |  | 1.29E-05 | Female |  |
| 753 | 3.43E-05 | Female |  | 1.30E-05 | Female |  |
| 754 | 3.80E-05 | Female |  | 1.31E-05 | Female |  |
| 755 | 3.84E-05 | Female |  | 1.33E-05 | Female |  |
| 756 | 3.94E-05 | Female |  | 1.35E-05 | Female |  |
| 757 | 3.98E-05 | Female |  | 1.37E-05 | Female |  |
| 758 | 3.57E-05 | Female |  | 1.37E-05 | Female |  |
| 759 | 3.67E-05 | Female |  | 1.39E-05 | Female |  |
| 760 | 3.54E-05 | Female |  | 1.39E-05 | Female |  |
| 761 | 3.50E-05 | Female |  | 1.40E-05 | Female |  |
| 762 | 4.33E-05 | Female |  | 1.41E-05 | Female |  |
| 763 | 3.78E-05 | Female |  | 1.42E-05 | Female |  |
| 764 | 3.61E-05 | Female |  | 1.43E-05 | Female |  |
| 765 | 3.82E-05 | Female |  | 1.43E-05 | Female |  |
| 766 | 3.96E-05 | Female |  | 1.44E-05 | Female |  |
| 767 | 3.79E-05 | Female |  | 1.45E-05 | Female |  |
| 768 | 3.95E-05 | Female |  | 1.46E-05 | Female |  |
| 769 | 4.00E-05 | Female |  | 1.47E-05 | Female |  |
| 770 | 3.23E-05 | Female |  | 1.48E-05 | Female |  |
| 771 | 4.08E-05 | Female |  | 1.48E-05 | Female |  |
| 772 | 4.09E-05 | Female |  | 1.52E-05 | Female |  |
| 773 | 4.22E-05 | Female |  | 1.52E-05 | Female |  |
| 774 | 6.23E-05 | Male | 1.19% | 1.58E-05 | Female |  |
| 775 | 4.75E-05 | Female |  | 1.58E-05 | Female |  |
| 776 | 4.46E-05 | Female |  | 1.60E-05 | Female |  |
| 777 | 3.96E-05 | Female |  | 1.61E-05 | Female |  |
| 778 | 4.52E-05 | Female |  | 1.62E-05 | Female |  |
| 779 | 3.82E-05 | Female |  | 1.65E-05 | Female |  |
| 780 | 4.04E-05 | Female |  | 1.66E-05 | Female |  |
| 781 | 4.27E-05 | Female |  | 1.66E-05 | Female |  |
| 782 | 4.36E-05 | Female |  | 1.68E-05 | Female |  |
| 783 | 3.23E-05 | Female |  | 1.69E-05 | Female |  |
| 784 | 3.94E-05 | Female |  | 1.69E-05 | Female |  |
| 785 | 4.09E-05 | Female |  | 1.70E-05 | Female |  |
| 786 | 4.15E-05 | Female |  | 1.70E-05 | Female |  |
| 787 | 4.50E-05 | Female |  | 1.74E-05 | Female |  |
| 788 | 4.34E-05 | Female |  | 1.76E-05 | Female |  |
| 789 | 4.66E-05 | Female |  | 1.77E-05 | Female |  |
| 790 | 4.22E-05 | Female |  | 1.77E-05 | Female |  |
| 791 | 4.20E-05 | Female |  | 1.77E-05 | Female |  |
| 792 | 4.81E-05 | Female |  | 1.83E-05 | Female |  |
| 793 | 4.45E-05 | Female |  | 1.94E-05 | Female |  |
| 794 | 4.49E-05 | Female |  | 1.99E-05 | Female |  |
